# The female upper reproductive tract harbors endogenous microbial profiles

**DOI:** 10.3389/fendo.2023.1096050

**Published:** 2023-06-21

**Authors:** Analuce Canha-Gouveia, Inmaculada Pérez-Prieto, Carmen Martínez Rodríguez, Teresa Escamez, Irene Leonés-Baños, Eduardo Salas-Espejo, Maria Teresa Prieto-Sánchez, Maria Luisa Sánchez-Ferrer, Pilar Coy, Signe Altmäe

**Affiliations:** ^1^ Department of Physiology, Faculty of Veterinary, University of Murcia, Murcia, Spain; ^2^ Biomedical Research Institute of Murcia (IMIB-Arrixaca), University Clinical Hospital "Virgen de la Arrixaca", Murcia, Spain; ^3^ Department of Biochemistry and Molecular Biology I, Faculty of Sciences, University of Granada, Granada, Spain; ^4^ Instituto de Investigación Biosanitaria ibs.GRANADA, Granada, Spain; ^5^ Genomics Unit, Biomedical Research Institute of Murcia (IMIB)-Arrixaca, Murcia, Spain; ^6^ BiobancMur-Nodo 1, Biomedical Research Institute of Murcia (IMIB)-Arrixaca, Murcia, Spain; ^7^ Spanish Biobank Platform, Carlos III Health Institute (ISCIII), Madrid, Spain; ^8^ Department of Obstetrics & Gynecology, “Virgen de la Arrixaca” University Clinical Hospital, Murcia, Spain; ^9^ Division of Obstetrics and Gynecology, Department of Clinical Science, Intervention and Technology (CLINTEC), Karolinska Institutet and Karolinska University Hospital, Stockholm, Sweden

**Keywords:** fallopian tubes, endometrium, 16S rRNA gene, microbes, microbiome, microbiota, upper reproductive tract

## Abstract

**Introduction:**

The female reproductive tract harbours unique microbial communities (known as microbiota) which have been associated with reproductive functions in health and disease. While endometrial microbiome studies have shown that the uterus possesses higher bacterial diversity and richness compared to the vagina, the knowledge regarding the composition of the Fallopian tubes (FT) is lacking, especially in fertile women without any underlying conditions.

**Methods:**

To address this gap, our study included 19 patients who underwent abdominal hysterectomy for benign uterine pathology, and 5 women who underwent tubal ligation as a permanent contraceptive method at Hospital Clínico Universitario Virgen de la Arrixaca (HCUVA). We analyzed the microbiome of samples collected from the FT and endometrium using 16S rRNA gene sequencing.

**Results:**

Our findings revealed distinct microbiome profiles in the endometrial and FT samples, indicating that the upper reproductive tract harbors an endogenous microbiome. However, these two sites also shared some similarities, with 69% of the detected taxa Being common to both. Interestingly, we identified seventeen bacterial taxa exclusively present in the FT samples, including the genera *Enhydrobacter, Granulicatella, Haemophilus, Rhizobium, Alistipes*, and *Paracoccus*, among others. On the other hand, 10 bacterial taxa were only found in the endometrium, including the genera *Klebsiella, Olsenella, Oscillibacter* and *Veillonella* (FDR <0.05). Furthermore, our study highlighted the influence of the endometrial collection method on the findings. Samples obtained transcervically showed a dominance of the genus Lactobacillus, which may indicate potential vaginal contamination. In contrast, uterine samples obtained through hysterescopy revealed higher abundance of the genera *Acinetobacter, Arthrobacter, Coprococcus, Methylobacterium, Prevotella, Roseburia, Staphylococcus*, and *Streptococcus*.

**Discussion:**

Although the upper reproductive tract appears to have a low microbial biomass, our results suggest that the endometrial and FT microbiome is unique to each individual. In fact, samples obtained from the same individual showed more microbial similarity between the endometrium and FT compared to samples from different women. Understanding the composition of the female upper reproductive microbiome provides valuable insights into the natural microenvironment where processes such as oocyte fertilization, embryo development and implantation occur. This knowledge can improve *in vitro* fertilization and embryo culture conditions for the treatment of infertility.

## Introduction

As our understanding of the human microbiota continues to expand, it becomes increasingly evident that it is ubiquitous and exerts significant influence on human physiology and pathophysiology (1–3). Within the female reproductive tract, a growing body of evidence is associating microbial composition to reproductive functions in both healthy and diseased states (4–7).

While numerous studies corroborate the important role of microbial communities in the female lower reproductive tract (vagina and cervix) in the defense against pathogens, the upper reproductive tract (endometrium, Fallopian tubes, ovaries) was traditionally considered a sterile environment, with the cervix acting as a barrier against bacterial passage (8). However, with the advent of microbiome studies focusing on the human upper reproductive tract and analysis of microbial genomes, it is now evident that this region possesses its own distinct microbial communities (7, 9, 10). Recent studies have consistently shown that the endometrium harbors greater bacterial diversity and richness compared to the lower reproductive tract. These microbial communities are mainly composed of bacteria belonging to the phyla *Firmicutes*, *Bacteroidetes* and *Proteobacteria*. The dominance of *Lactobacillus* in the uterus has been associated with a higher probability of live births, while the presence of *Gardnerella* or *Streptococcus* has been linked to early pregnancy loss or implantation failure in IVF treatment (1, 11). However, due to differences in study design and the absence of proper negative and positive controls, there is a lack of consensus among studies examining the upper reproductive tract microbiota (9, 12).

The microbial composition of the Fallopian tubes (FT) is less studied, primarily due to challenges associated with sample collection which may affect future fertility. The characterization of the endogenous microbiome of the FT is of particular interest because this microenvironment provides a stable temperature, optimal pH and dynamic fluid secretions that support oocyte fertilisation and the early stages of embryo development (13–15). The limited studies analysing samples from women with benign diseases or for prophylactic purposes suggest that the FT does indeed harbor an endogenous microbiome. Predominant bacterial taxa identified in these studies include *Firmicutes* (especially *Staphylococcus* sp., *Enterococcus* sp., and *Lactobacillus* sp.), *Pseudomonas* sp. *Burkholderia* sp., *Propionibacterium* sp. and *Prevotella* sp (15–18). However, there is ongoing debate regarding whether the FT truly harbors an endogenous microbiome and to what extent it impacts oocyte fertilisation and the initial stages of embryo development.

Given the anatomical connection between the uterus and the FT, with the intramural portion of the uterine tube preventing a complete physical separation between the two sites, it is reasonable to hypothesize that the microbiome of the FT may be similar to that of the uterus (19–21). Therefore, comparative studies analyzing uterine and FT samples collected simultaneously from the same donor are necessary to evaluate whether the organs comprising the female upper reproductive tract possess specific endogenous microbial profiles. In the current study, we aimed to analyse the 16S rRNA gene V2-4 and V6-9 regions of endometrial and FT samples obtained from fertile women, with the objective of identifying the microbiome of the female upper reproductive tract in disease-free individuals.

## Materials and methods

### Study population

**Figure 1:**
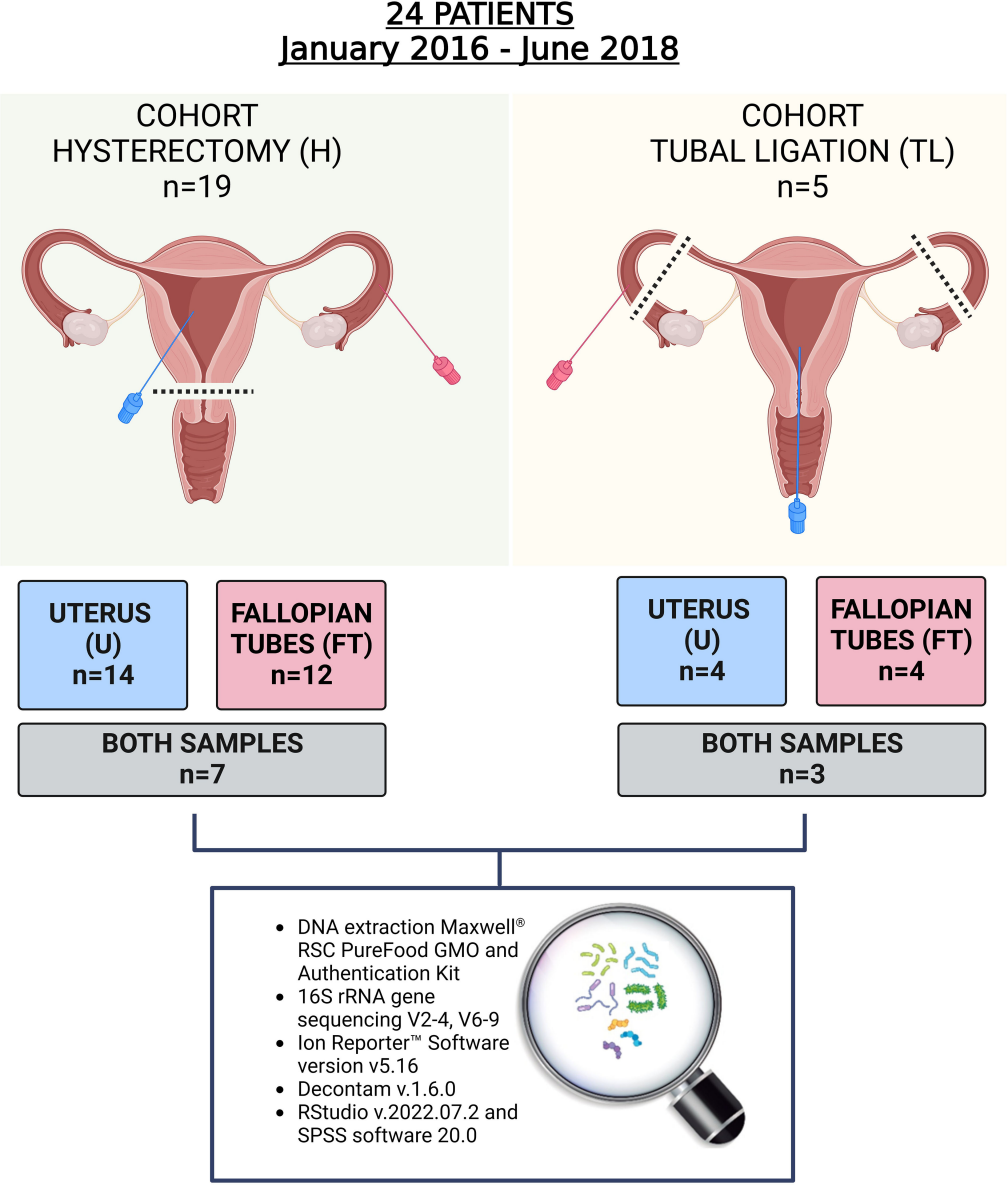
Study design. In total 24 women participated in the study and 34 samples from the upper reproductive tract were retrieved. In the hysterectomy cohort (H), seven women provided both endometrial and Fallopian tube (FT) samples, and in the tubal ligation cohort (TL), three women provided both samples. The rest of the participants provided only one of the samples due to the tissue damage during laparoscopic procedure, non-sterile condition, or blood contamination.

This prospective studywas conducted at the Service of Obstetrics and Gynaecology of the HCUVA in Murcia, Spain. Patients who underwent a planned laparoscopic hysterectomy with bilateral salpingo-oophorectomy or laparoscopic tubal ligation from January 2016 until June 2018 were recruited to participate in the study. Inclusion criteria were as follows: Caucasian women who had not received hormonal treatment for three months prior to surgery, regular menstrual cycles, and no history of fertility problems, endometriosis or other adnexal pathology detected by transvaginal ultrasound analysis and confirmed through histological examination. Nineteen participants underwent total laparoscopic hysterectomy with bilateral salpingo-oophorectomy to remove the uterus, cervix, ovaries, and FT due to the presence of uterine fibroids and associated abnormal bleeding (see **Figure 1** for the study design). Additionally, five participants underwent tubal ligation to remove the FTs as a permanent contraception/sterilization measure. This study was approved by the Ethics Research Committee (CEIC) of HCUVA, Murcia, Spain (Approval No. EST: 04/16) and all participants provided written informed consent. Patient data and samples included in this study were registered, stored, and processed by the Biobanco en Red de la Región de Murcia, BIOBANC-MUR (registered on the Registro Nacional de Biobancos – ISCIII, no. B.0000859).

### Collection of FT and endometrial samples

The collection method for the FT samples was standardizedfor all patients who underwent laparoscopic hysterectomy with bilateral salpingo-oophorectomy or laparoscopic tubal ligation. After the laparoscopic procedure, FTs were removed and transferred to ice-cold Petri dishes. Once dissected, FTs were clamped at both opposite ends to avoid sample waste. Manual mechanical pressure was applied between the extremities, the FT content that accumulated at the upper portion of the ampulla was aspirated through a sterile Mucat device (CDD Laboratorie, France). This class I medical device, complying with Directive 93/42/EEC, indicated for direct exocervical or endocervical aspiration and Hühner test, was adapted to be easily introduced into the tubes. Once introduced, aspiration of the content was performed with the integrated plunger, which slides up and down when pushed by a flexible acetal resin shaft, without a syringe. The content was immediately aliquoted in 1,5ml Eppendorf Safe-Lock^®^ Tubes, frozen in liquid nitrogen until further analysis.

For the endometrial samples, the collection method avried depending on the type of surgery. During hysterectomy, when the entire upper reproductive tract was removed, direct access to the uterus was achieved using a sterile Mucat device (CDD Laboratorie, France). The device was carefully maneuvered to avoid sampling uterine fibroid tissue (clearly identified visually), as well as potential microbial contamination from the vagina or the cervix. In contrast, for patients undergoing tubal ligation without uterus removal, a speculum was inserted to gently separate the vagina, allowing visualization of the cervix. The cervix was cleaned with sterile saline solution and then the sterile Mucat device (CDD Laboratorie, France) was inserted into the cervix to reach the interior of the uterus. The aspiration of the uterine content was performed with the integrated plunger as previously described (22). The collected content was stored in 1,5ml Eppendorf Safe-Lock^®^ Tubes, and frozen in liquid nitrogen until further analysis.

### DNA extraction, amplification, library preparation, and sequencing

DNA extraction from the stored samples was performed using the Maxwell^®^ RSC PureFood GMO and Authentication Kit and Maxwell^®^ RSC Equipment (Promega, USA). A NanoDrop spectrophotometer was used to determine the DNA yield (A260) and purity (A260/A280 ratio) (**Supplementary Table 1**).

Bacterial identification was performed by Genomics Unit from Institute for Biomedical Research of Murcia IMIB-Arrixaca. The multiplex PCR using Ion Torrent 16S Metagenomics kit (Thermo Fisher Scientific Inc., **USA)** was used to amplify the 16S rRNA gene.Two sets of primers to target the regions V2, V4, V8, and V3, V6-7, V9 (**Supplementary Table 2**). Amplification was performed in a SimpliAmp thermal cycler (Applied Biosystems, USA) following the program: denaturation at 95°C for 10 min, followed by a cyclic 3-step stage consisting of 25 cycles of denaturation at 95°C for 30 s, annealing at 58°C for 30 s, and extension at 72°C for 20 s; at the end of this stage, the program concluded with an additional extension period at 72°C for 7 min and the reaction was stopped by cooling at 4°C. The resulting amplicons were tested by electrophoresis using 2% agarose gel in tris-acetate-EDTA (TAE) buffer, purified with AMPure^®^ XP Beads (Beckman Coulter Inc., USA), and quantified using QubitTM dsDNA HS Assay Kit in a Qubit 3 fluorometer (Invitrogen, Thermo Fisher Scientific Inc., **USA**). Afterwards, the Ion Plus Fragment Library Kit (Thermo Fisher Scientific Inc., **USA)** was used to generate a library from each sample. Each library was indexed by ligating Ion Xpress ™ Barcode Adapters (Thermo Fisher Scientific Inc. **USA)** to the amplicons. Libraries were purified with AMPure^®^ XP Beads and quantified using the Ion Universal Library Quantitation Kit (Thermo Fisher Scientific Inc., **USA)** in a QuantStudio 5 Real-Time PCR Instrument (Applied Biosystems, USA).

Next, the libraries were pooled and clonally amplified onto Ion Sphere Particles (ISPs) by emulsion PCR in an Ion OneTouch™ 2 System (Thermo Fisher Scientific Inc., **USA)** according to the manufacturer´s instructions. Sequencing of the amplicon libraries was carried out on an Ion 530™ Kit (Thermo Fisher Scientific Inc. **USA**) on an Ion S5™ System (Thermo Fisher Scientific Inc., **USA)**.

### Data processing

After sequencing, the individual sequence reads were filtered by the Torrent Suite ™ Software v5.12.1 to remove the low quality and polyclonal sequences. The quality filtered data were analyzed using Ion Reporter™ Software version v5.16. Clustering into operational taxonomic units (OTUs) and taxonomic assignment were performed based on the Basic Local Alignment Search Tool (BLAST) using two reference libraries, MicroSEQ^®^ 16S Reference Library v2013.1 and the Greengenes v13.5 database (Life Technologies Corporation, USA). For an OTU to be accepted as valid, at least ten reads with an alignment coverage ≥ 90% between the hit and query were required. Identifications were accepted at the genus level with sequence identity > 97%.

Given that characterization of the low microbial biomass site like the upper reproductive tract requires meticulous contamination control, *in-silico* decontamination approach using Decontam v.1.6.0 (23, 24) was applied to discern between the true bacterial sequences and potential contaminants. To use this method, a table of the relative abundances of OTUs (columns) in each sample (rows) was created from the raw data. Next, we included DNA concentration of each sample in the model (from **Supplementary Table 1**). The Decontam score threshold was set to 0.1 as a default setting to define contaminating phylotypes (23). The relative abundance of the considered contaminant phylotypes was set to zero as described previously (24). Furthermore, for diversity and abundance analyses we additionally filtered out those taxa that were detected in less than 30% of the remaining samples, as previously described (25).

### Statistical analyses

Statistical analyses were performed using the R statistical software v.4.2.1 under RStudio v.2022.07.2 and SPSS software 20.0 (SPSS, USA). Microbiome data were aggregated to genus level for diversity and abundance comparisons. All relative abundances were expressed as median and first and third quartiles (q1, q3). Normal distribution of the variables was tested by using the Shapiro-Wilk test. Relative abundances of identified genera did not meet normality and were analyzed using the nonparametric Mann-Whitney *U* test. Furthermore, the Analysis of Compositions of Microbiomes with Bias Correction (ANCOM-BC) was performed to validate our results. Benjamini-Hochberg method (false discovery rate [FDR]) was used to obtain adjusted p-values in multiple comparisons. Differences were considered statistically significant between groups when p < 0.05. Alpha-diversity indices (Shannon diversity index and OTUs number [i.e., richness]) were calculated using the diversity function of the *vegan* R package, both in FT and endometrial samples. Differences among the groups of samples’ diversity indices were tested using Mann-Whitney *U* test. Additionally, alpha-diversity was compared between women with both types of samples using a Wilcoxon signed-rank test for paired data. Bray-Curtis dissimilarity was calculated using *vegdist* R function and Permutational Analysis of Variance (PERMANOVA) was performed to analyze beta-diversity using *adonis* R function.

## Results

### Samples

A total of 34 samples were collected from 24 enrolled patients. The patients’ characteristics are presented in **Table 1** and **Supplementary Table 3**. As indicated in **Figure 1**, from the group that underwent laparoscopic tubal ligation, four FT samples and four transcervical endometrial samples were collected. In the hysterectomy group, which involved the extraction of the upper reproductive tract, 12 FT samples and 14 endometrial samples were obtained from the uterus, avoiding uterine fibroid tissue. It was not always possible to collect both types of samples from each patient because some anatomical pieces were damaged after being removed by laparoscopic techniques, and due to the impossibility of collecting some samples with the required sterile conditions and without blood contamination. Both FT and endometrial samples were successfully collected from seven out of 19 patients of the hysterectomy cohort, while three out of the five patients in the tubal ligation cohort provided both samples (**Figure 1**).

**Table 1:** Demographic characteristics (age, body mass index- BMI and parity) of the study population and collected samples from two groups of patients: patients who underwent a total laparoscopic hysterectomy with bilateral salpingo-oophorectomy and patients who submitted to a laparoscopic tubal ligation.

Study Population Groups	Hysterectomy n=19	Tubal ligation n=5
Age (years)	45 ± 3	37 ± 4
BMI	28,5 ± 4,7	28,3 ± 4
Parity	1,8 ± 0,9	2,2 ± 0,5
Fallopian tube samples	12	4
Endometrial samples	14	4
Both tissue samples	7	3

### Data processing

A total of 245 and 252 bacterial genera were identified in the endometrial and FT samples, respectively. The average number of reads per FT sample was 25241,44 ± 10845,46 (mean ± SD). For the endometrial samples, the average number of reads per sample was 30845 ± 18702,56 (mean ± SD). Applying the decontamination method using Decontam, two genera, *Aerococcus* in FT samples and *Acidovorax* in the endometrial samples, were identified as contaminant phylotypes and removed from the analysis. Furthermore, to ensure the identification of the “core” microbiome of both sites, an additional filtering step was applied, eliminating bacterial taxa present in less than 30% of the participants, as previously described (25). As a result, a total of 77 bacterial genera were identified in the FT samples (**Supplementary Table 4**), and 70 bacterial genera were identified in the endometrial samples (**Supplementary Table 5**).

### Microbial profiles of FT samples

**Figure 2:**
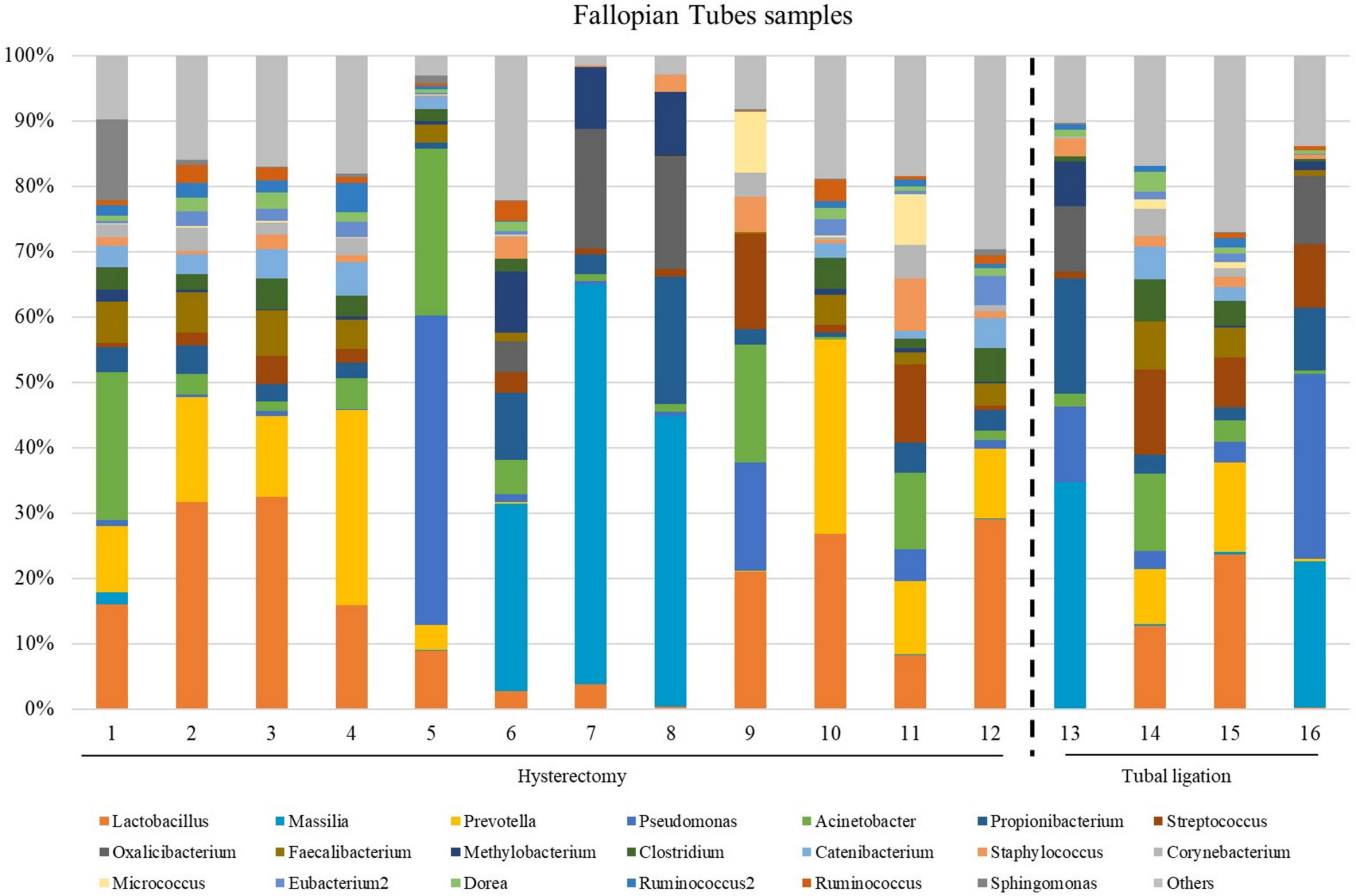
The most abundant genera detected in the Fallopian tubes (FT) samples from patients underwent atotal laparoscopic hysterectomy with bilateral salpingo-oophorectomy (patients 1, 2, 3, 5, 9, 10, 11, 12, 13, 15, 16 and 17) or laparoscopic tubal ligation (patients 20, 21, 23 and 24). Percent-stacked barchart of those genera whose mean relative abundances were higher than 1% are represented.

The microbial composition at the genus level in FT samples exhibited variability across different samples (**Figure 2**; **Supplementary Figure 1** at family taxonomic level). The most abundant genera among all samples were *Lactobacillus* (relative abundance =14.3 [3.48;24.4]), *Prevotella* (relative abundance = 9.29 [0.31;12.7]), *Acinetobacter* (relative abundance =3.20 [1.36;11.7]), *Propionibacterium* (relative abundance =3.09 [2.45;5.86]) and *Faecalibacterium* (relative abundance =3.09 [0.68;4.97]) (**Supplementary Table 4**).

Since the fertile women undergoing tubal ligation had no associated pathology, while women undergoing hysterectomy were diagnosed with benign uterine fibroids, a comparative microbiome analysis was performed to investigate any potential influence of uterine fibroids on the microbial microenvironment in the tubes. No significant differences were revealed in microbial diversity, or in the differential abundance analysis between the two groups (**Supplementary Table 6**).

### Microbial profiles of endometrial samples

**Figure 3:**
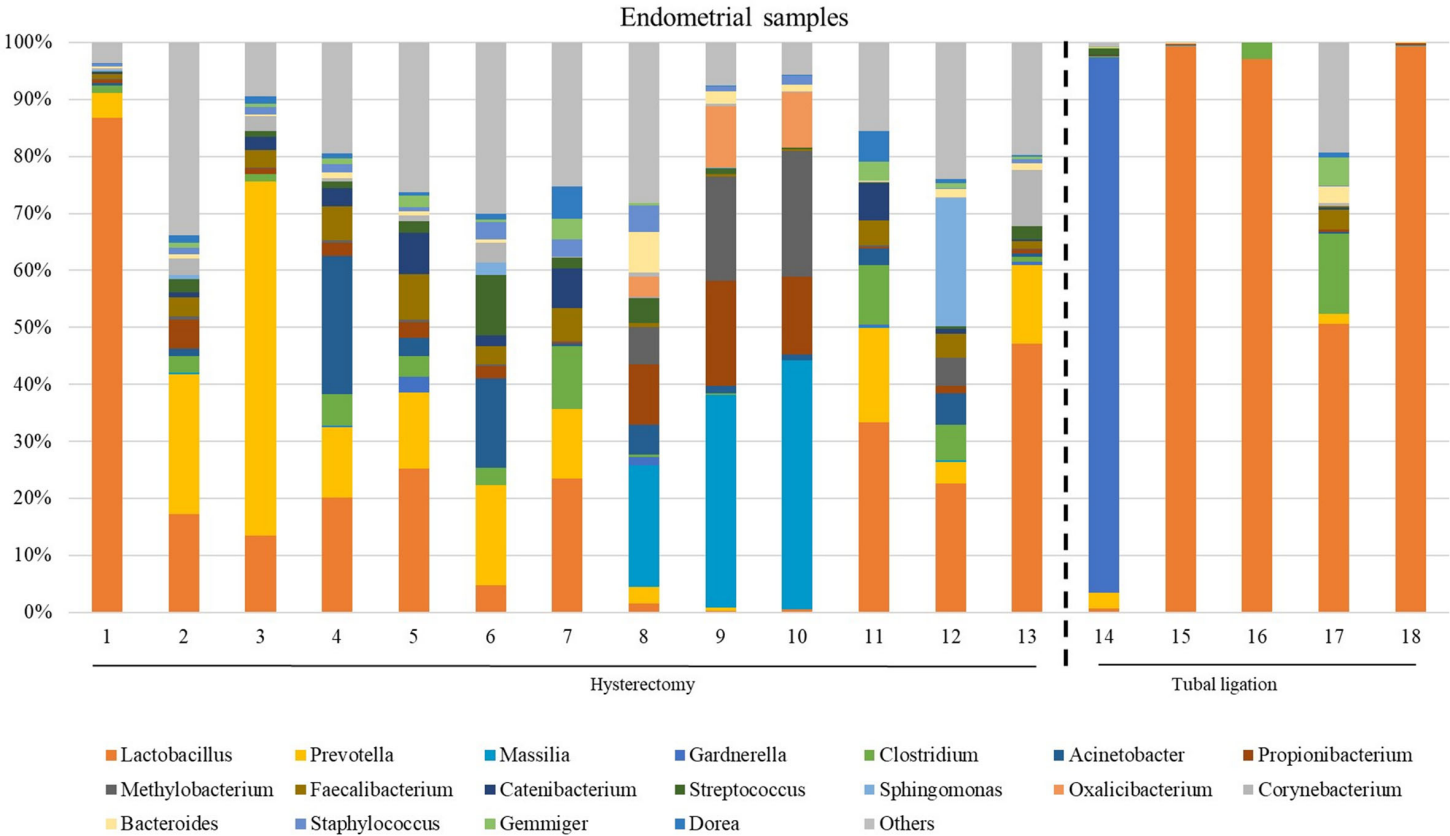
The most abundant genera detected in the endometrial samples from patients undergoing a total laparoscopic hysterectomy with bilateral salpingo-oophorectomy (patients 1, 2, 3, 4, 6, 7, 8, 10, 12, 13, 14, 15, 18 and 19) or laparoscopic tubal ligation (patients 20, 22, 23 and 24). Percent-stacked barchart of those genera whose mean relative abundances were higher than 1% are represented.

The microbiome composition revealed heterogeneity among the endometrial samples. The genus *Lactobacillus* showed the highest average abundance (relative abundance =23.0 [6.89;49.8]), followed by *Prevotella* (relative abundance =4.13 [0.85;13.7]), *Faecalibacterium* (relative abundance =2.18 [0.24;4.12]), and *Clostridum* (relative abundance =2.08 [0.32;5.06]) (**Figure 3**; **Supplementary Table 5**, and **Supplementary Figure 2** indicating family taxonomic level).

**Figure 4:**
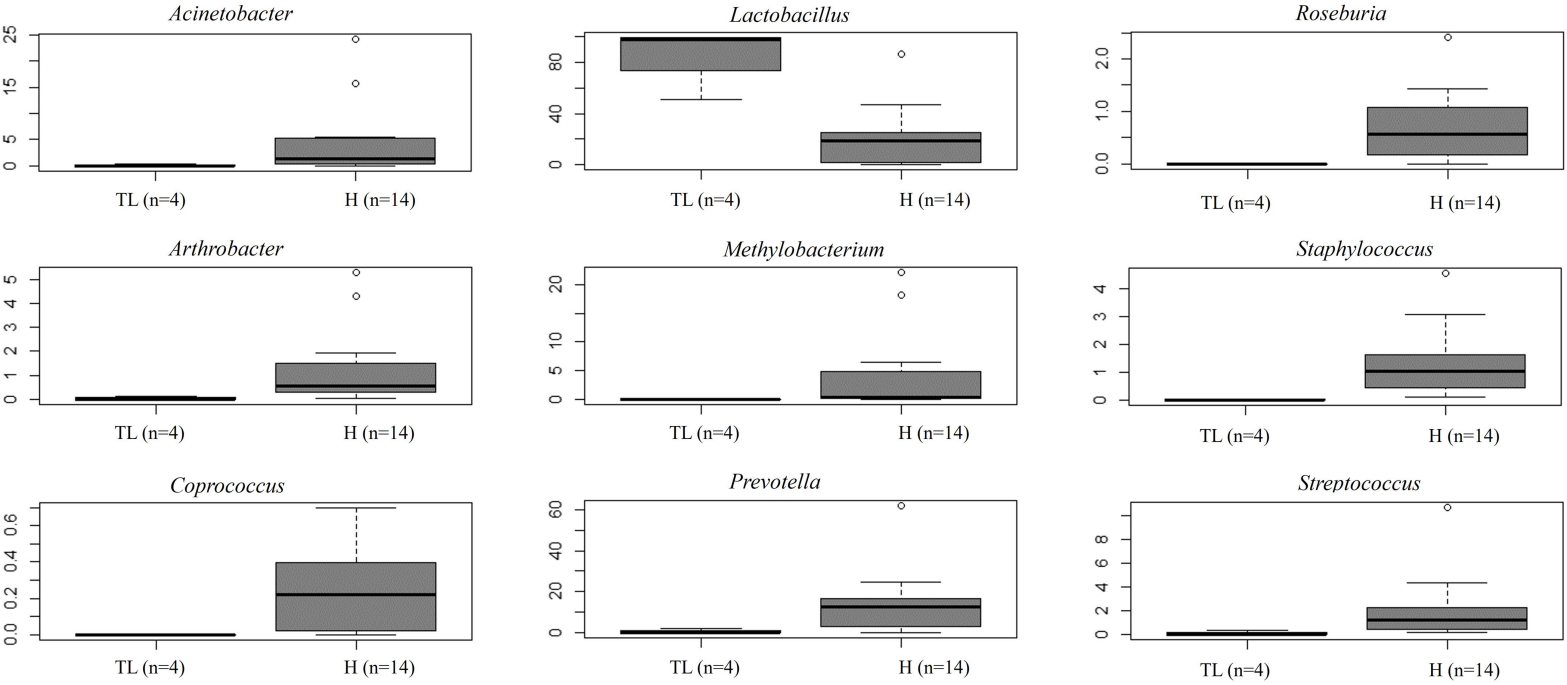
Relative abundance of nine bacterial genera between samples obtained directly from the uterus (hysterectomy, H) (fertile women with fibroids) and transcervically when undergoing tubal ligation (TL) (fertile women without the disease). After multiple testing correction adjustment, the difference remained marginal (FDR=0.083 for all plots).

Unlike FT samples, the collection method for endometrial samples varied depending on the surgical procedure. In patients undergoing hysterectomy for benign uterine conditions, the entire upper reproductive tract was extracted, allowing direct access to the uterine cavity without passing through the vaginal and cervical canal. The fibroid tissue was visually identified and biopsied, focusing on tissue that presented unaltered morphological characteristics. On the other hand, in women undergoing to tubal ligation for contraceptive purposes and without underlying disease, endometrial biopsy was obtained transcervically. Therefore, we aimed to compare whether the uterine microenvironment could be influenced by the fibroids and whether the sampling method *via* cervix (high bacterial contamination risk) could have an impact on the microbial composition in the endometrial samples. When comparing the microbiome of the two sampling techniques, 20 genera presented significantly different abundance (**Supplementary Table 7**). When applying the multiple testing correction, nine genera remained as marginally different between the groups, where *Lactobacillus* was more abundant while *Acinetobacter, Arthrobacter, Coprococcus, Methylobacterium, Prevotella, Roseburia, Staphylococcus, Streptococcus* were less abundant in samples obtained transcervically (**Figure 4**; **Supplementary Table 7**).

### Microbiome composition between endometrial and FT samples

**Figure 5:**
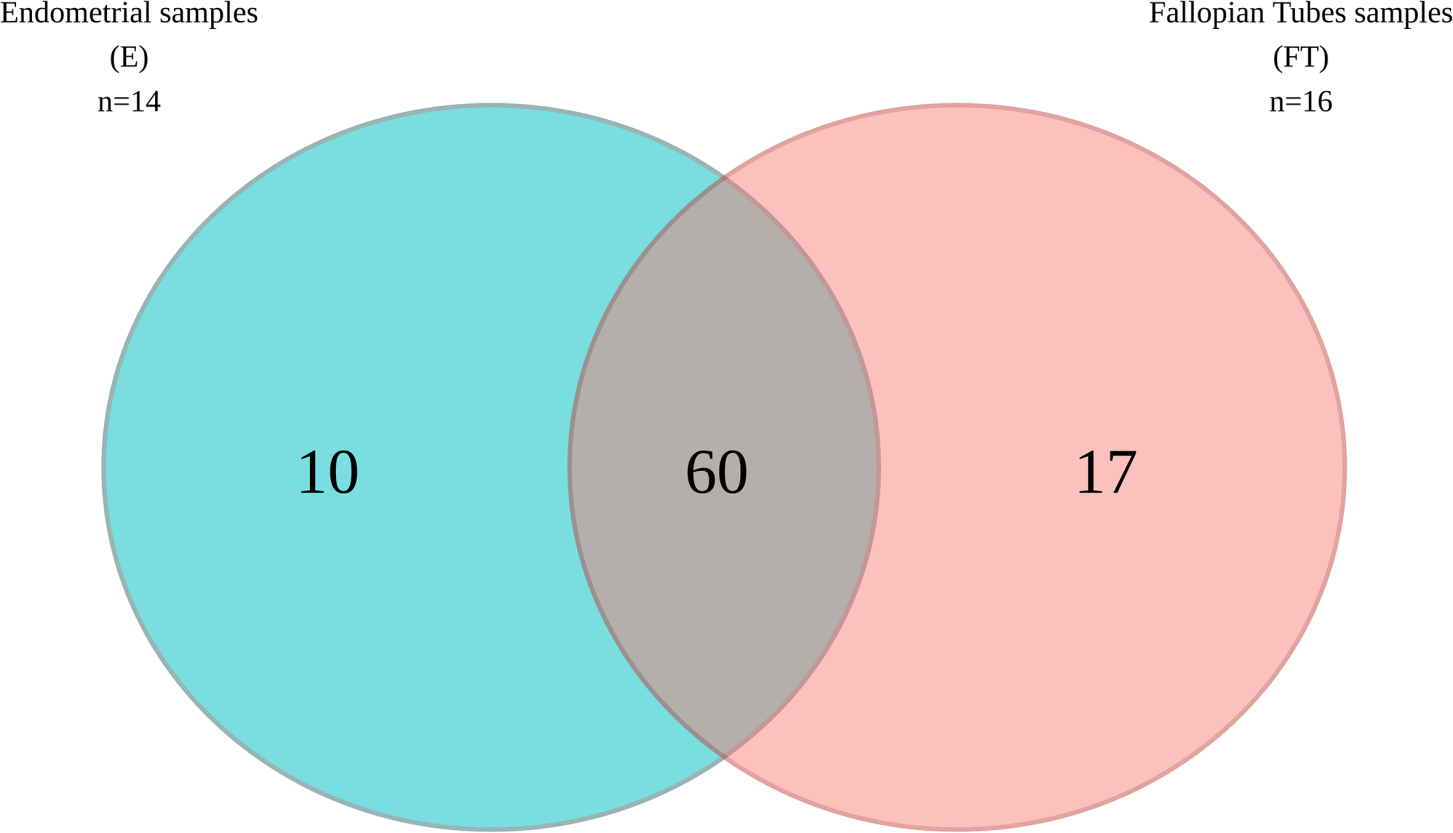
Venn diagram illustrating the bacterial genera present in the upper reproductive tract.

When comparing microbial composition between the endometrium and FT, the endometrial samples from the tubal ligation group were excluded f4rom the analysis. This decision was made due to significant microbiome differences, potentially indicating vaginal orcervical contamination characterized by a high abundance of *Lactobacillus*). Thus, 16 FT samples and 14 endometrial samples were compared. A considerable portion of the detected taxa (60 genera) was found in both sites, indicating shared microbial composition. Additionally, 17 bacterial genera were exclusively detected in the FT samples, while 10 genera were considered endometrial-specific (**Figure 5**; **Table 2**). Out of these detected genera (**Supplementary Table 8**), the relative abundance of 11 genera was significantly between uterine and FT samples, as confirmed by both the Mann-Whitney U test and ANCOM-BC analysis. Specifically, *Gardnerella* (p=0.002; FDR=0.042), *Klebsiella* (p=0.004; FDR=0.042), *Olsenella* (p=0.004; FDR=0.042), *Oscillibacter* (p=0.004; FDR=0.042) and *Veillonella* (p=0.004; FDR=0.042) were found to be more prevalent in the endometrium.Conversely, *Enhydrobacter* (p=0.001; FDR=0.042), *Granulicatella* (p=0.001; FDR=0.042), *Haemophilus* (p=0.003; FDR=0.042), *Rhizobium* (p=0.003; FDR=0.042), *Alistipes* (p=0.006; FDR=0.048) and *Paracoccus* (p=0.006; FDR=0.048) were more abundant in FT samples (p values obtained from the strict Mann-Whitney U test analysis).

**Table 2:** Microbial composition of the endometrial and Fallopian tube (FT) samples.

Fallopian tubes	Fallopian tubes and Endometrium			Endometrium
*Aeromonas* †	*Acinetobacter*	*Actinomyces*	*Anaerococcus*	*Barnesiella* †
Alistipes*†	*Arthrobacter*	*Bacillus*	*Bacteroides*	*Brachymonas*†
*Bifidobacterium* †	*Bilophila*	*Blautia*	*Butyricimonas*	*Chryseobacterium*†
*Brachyspira*†	*Campylobacter*	*Catenibacterium*	*Cloacibacterium*	Gardnerella*†
*Brevundimonas*†	*Clostridium*	*Collinsella*	*Coprococcus*	Klebsiella*†
*Burkholderia* †	*Corynebacterium*	*Desulfovibrio*	*Dialister*	Olsenella*†
*Comamonas* †	*Dolosigranulum*	*Dorea*	*Enterococcus*	Oscillibacter*†
Enhydrobacter*†	*Eubacterium*	*Eubacterium2*	*Faecalibacterium*	*Serratia* †
*Flavonifractor* †	*Finegoldia*	*Gemella*	*Gemmiger*	Veillonella*†
*Fusobacterium* †	*Helicobacter*	*Herbaspirillum*	*Kocuria*	*Vibrio*†
Granulicatella* †	*Lachnoclostridium*	*Lactobacillus*	*Lactococcus*	
Haemophilus*†	*Massilia*	*Megasphaera*	*Methylobacterium*	
Paracoccus*†	*Microbacterium*	*Micrococcus*	*Mitsuokella*	* *
*Parasutterella* †	*Moraxella*	*Neisseria*	*Oxalicibacterium*	* *
Rhizobium*†	*Parabacteroides*	*Pelomonas*	*Phascolarctobacterium*†	* *
*Shewanella* †	*Porphyromonas*	*Prevotella*	*Propionibacterium*	* *
*Sutterella* †	*Pseudoflavonifractor*	*Pseudomonas*	*Ralstonia*	* *
* *	*Roseburia*	*Rothia*	*Ruminiclostridium*	* *
* *	*Ruminococcus*	*Ruminococcus2*	*Sphingomonas*	* *
* *	*Staphylococcus*	*Streptococcus*	*Subdoligranulum*	* *

The asterisks (*) represent the differentially abundant microbial taxa between uterine and FT samples analyzed by the non-parametric Mann-Whitney U test (p<0.05). The crosses (†) represent the differentially abundant microbial taxa between the endometrial and FT samples analyzed by the Analysis of Compositions of Microbiomes with Bias Correction (ANCOM-BC) (p<0.05). P-values were adjusted for the multiple testing correction (False Discovery Rate, FDR).

**Figure 6:**
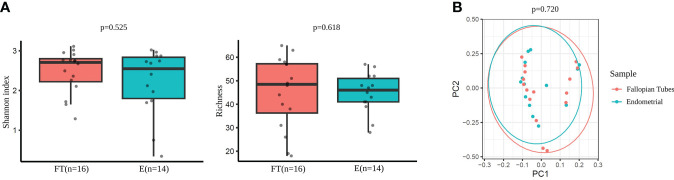
Diversity indices in Fallopian tubes (FT) and endometrial (E) samples. **(A)** Alpha-diversity metrics (i.e., Shannon, OTUs number [richness]) of endometrial and FT samples. **(B)** Beta-diversity represented by principal coordinate analysis (PCoA) based on Bray-Curtis distances (PERMANOVA, R2=0.024, p=0.720) between endometrial and FT samples.

No significant differences were detected between the endometrial and FT samples in alpha-diversity metrics when comparing the microbiome diversity of endometrial and FT samples (i.e., Shannon, OTUs number [richness]) (**Figure 6A**). Also, beta-diversity represented by PCoA blot based on Bray-Curtis distances did not show any significant dissimilarities between the microbiome composition between the two sample types (**Figure 6B**).

### Sensitivity analysis in paired endometrial and FT samples

A sensitivity analysis was performed using samples exclusively from patients who underwent hysterectomy (n=7) and had valid samples from both tissues (endometrium and FT) (**Figure 1**; **Supplementary Table 3**). This approach aimed to avoid the possible contamination effect from cervical bacteria.

**Figure 7:**
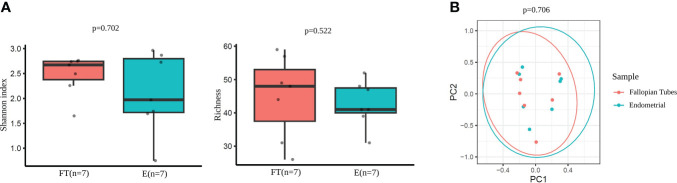
Diversity indices in paired endometrial and FT samples. **(A)** Alpha-diversity metrics (i.e., Shannon, OTUs number [richness]) of endometrial and FT samples when the restricted group of patients with paired samples was selected. **(B)** Beta-diversity represented by principal coordinate analysis (PCoA) based on Bray-Curtis distances of patients with paired samples (PERMANOVA, R2 = 0.048, p=0.706).

The comparison of microbial diversity between endometrial and FT samples revealed no significant differences in alpha- (**Figure 7A**) and beta- (**Figure 7B**) diversity metrics. (p >0,05).

In this more restricted subset of samples, the previously observed statistical differences in the relative abundances of the 11 genera (*Gardnerella, Klebsiella, Olsenella, Oscillibacter, Veillonella, Enhydrobacter, Granulicatella, Haemophilus, Rhizobium, Alistipes and Paracoccus*) between endometrial and FT samples did not remain statistically significant after adjusting for the multiple testing correction (FDR) (**Supplementary Table 9**).

**Figure 8:**
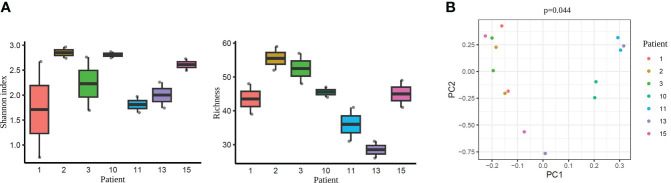
Diversity indices each pair of the tissue samples corresponding to their respective patient. **(A)** Alpha-diversity metrics (i.e., Shannon, OTUs number) of paired endometrium and Fallopian tube (FT) samples from the same women (n=7), all values p >0.05. Each label indicates a patient (e.g. 1). **(B)** Beta-diversity represented by principal coordinate analysis (PCoA) based on Bray-Curtis distances of patients with paired samples (PERMANOVA, R^2 = ^0.622, p=0.044). Each patient is indicated with one colour, where the two dots of the same colour represent individuals’ endometrial and FT samples.

As a next step, we performed an additional comparison considering each pair of samples from the same patient. Alpha-diversity analysis did not detect any statistically significant differences when comparing the paired tissue samples of each patient (Shannon diversity index and OTUs number with p >0.05; **Supplementary Tables 10**, **11**, respectively) (**Figure 8A**). However, beta-diversity analysis revealed a significant dissimilarity when comparing the paired samples from the same woman (PERMANOVA, p=0.044) (**Figure 8B**). This finding suggests that the microbiome within an individual, even from two different tissue types (endometrium and FT), is more similar than the same tissue type (e.g. endometrium) between different individuals.

## Discussion

The female upper reproductive tract plays a critical role in oocyte fertilisation, early embryo development, and embryo implantation. Understanding the detailed microenvironment in the FT and endometrium is essential for manipulating and improving conditions in assisted reproduction technologies. Over 20% of couples at reproductive age suffer infertility, and with the socioeconomic situation where couples delay family planning and have children later in life, the demand for infertility treatment continues to rise worldwide (26).

There is a growing awareness that the microbes colonizing our body are involved in various pathological processes. Therefore, studying the microbiome of female reproductive tract has become a hot topic in order to understand its role in crucial events such as embryo development and pregnancy establishment (6). Imbalances in the uterine cavity microbiome have been associated with implantation failure, decreased success of assisted reproductive technologies, as well as conditions like endometriosis, endometritis, polyps, and endometrial cancer (10, 27, 28). However, very few studies have analysed FT microbiome due to ethical and technical challenges associated with obtaining FT sample without compromising future fertility. As a result, there is currently no consensus on the core microbial composition of the upper reproductive tract, whether in healthy or pathological conditions (10, 12, 29–31), and further research is needed.

The current study analysed the microbial composition of the upper reproductive tract in women with confirmed fertility. We examined FT and endometrial samples from patients diagnosed with benign uterine pathology or without the disease. Our findings revealed a shared (~70%) endogenous microbial community present in both sites of the upper reproductive tract, whith *Lactobacillus*, *Prevotella*, and *Faecalibacterium* being the most prevalent taxa. Considering that the intramural portion of the uterine tube in humans does not allow for physical separation between the FT and uterine environments, it is reasonable to assume that there is smooth communication between these anatomical regions, resulting in similar microbiomes. We detected 60 bacterial genera common to both tissues, while 17 bacterial genera were FT-specific and 10 were uniquely present in the endometrium. *Gardnerella*, *Klebsiella*, *Olsenella*, *Oscillibacter*, and *Veillonella* were significantly associated with the endometrial samples, while *Enhydrobacter*, *Granulicatella*, *Haemophilus*, *Rhizobium*, *Alistipes*, and *Paracoccus* were more abundant in FT samples. Although the presence of these genera in the upper reproductive tract has been previously described (8, 16, 32), the site specificity demonstrated in our results has not been reported before.

When comparing the FT and endometrial samples obtained from the same women, although the sample size was limited, it seems that the two distinct tissue microbiomes were more similar within an individual than the same tissue sample between different individuals. These data support the hypothesis that each person has their own “microbial fingerprint”, with microbial residents tailored to their environmental conditions – namely their genetics, diet, and developmental history. These residents persist over time and help to defend against invaders (33). So, it is expected that there would be more microbial similarities between different body sites within an individual compared to specific body sites between different individuals. Similar results have been described previously,although with more heterogenous cohorts (18). Thus, establishing a ‘core’ microbiome becomes challenging, as what might be considered healthy in one person may differ from another, adding complexity to the investigation of the human microbiome.

Our study included fertile women with benign uterine conditions (fibroids) and women without the disease who underwent tubal ligation as a terminal contraceptive method. This led to two different methods for obtaining study material: hysterectomy and tubal ligation. The study evaluated the effect of fibroids-related uterine microenvironment on the FT microbiome. FT samples were obtained in both cohorts using the same method, allowing us to study this effect. Our findings showed no association between the fibroids-free endometrial microbiome from women with uterine fibroids and the microbiome of FT. This suggests that fibroids-related uterine environment does not seem to affect the FT microenvironment.

In contrast, the sampling method for obtaining endometrial samples differed considerably between the two cohorts.: In the hysterectomy cases, the reproductive organs were removed, and the endometrial samples were obtained directly by opening the uterus under sterile conditions. However, in the tubal ligation cases, the endometrial samples were obtained transcervically, posing a higher risk of bacterial contamination from the lower reproductive tract (vagina/cervix). Thus, when analysing the endometrial samples from these two cohorts, we cannot determine whether the significant differences observed in the endometrial microbial composition are due to the fibroids-associated uterine microenvironment or the sampling method itself. After applying multiple testing correction, nine genera remained marginally different between the groups. *Lactobacillus* was more abundant in samples obtained transcervically, while *Acinetobacter, Arthrobacter, Coprococcus, Methylobacterium, Prevotella, Roseburia, Staphylococcus, Streptococcus* were more abundant in hysterectomy samples. The difference in *Lactobacillus* abundance depending on the sampling method has been previously reported, with lower dominance linked to surgeries carrying a lower contamination risk from the vagina and cervix, such as hysterectomy (30), laparoscopy (8) and/or cesarean section (34) (10). In line with these studies, the uterine samples collected transcervically in our study showed a clear dominance of *Lactobacillus* (abundance of 98,2%), while samples obtained during hysterectomy showed higher diversity and lower prevalence of *Lactobacillus* (abundance of 18,7%). Based on these findings, we believe that the sampling method had a stronger effect on the endometrial microbiome than the fibroids-free uterine sample. A previous study by Winters et al. reported that the endometria of women with a median age of 45, who underwent hysterectomy for fibroids were dominated by *Acinetobacter* (abundance of 60%) (30). Other studies have suggested that *Acinetobacter* may be associated with a normal (or benign) endometrium, while *Methylobacterium* has been associated with endometrial cancer (35). In our study, disease-free endometrial samples from women with uterine fibroids showed a small relative abundance of *Acinetobacter* and *Methylobacterium*. These two genera, however, along with *Arthrobacter, Coprococcus, Prevotella, Roseburia, Staphylococcus*, and *Streptococcus*, which showed differential presence in endometrial samples, are considered common contaminant genera (9). Therefore, further research is required to determine which genera are contaminant and which have a role in uterine health. This could involve enrichment analysis of metabolic pathways using RNAseq analysis or whole metagenomics analysis, as well as investigating the impact of factors like uterine fibroids and other pathologies on the microbial composition. Interestingly, a recent study has associated *Clostridium, Ruminococcus, Blautia* and *Lactobacillus* (which were found in both tissues in our study) with Tryptophan metabolism (12). This suggests a potential host–microbiota crosstalk in the biosynthesis of serotonin and melatonin, as well as serotonin degradation, where Tryptophan acts as a precursor. Specifically, dysregulation of melatonin has been linked to altered uterine functions, including endometrial receptivity and recurrent spontaneous abortion (36).

Our study is the first to analyze the endometrial and FT samples together from women with confirmed fertility. Nevertheless, some limitations should be acknowledged. Firstly, the relatively small sample size makes the study results preliminary and highlight the need for confirmation in a larger sample size. Secondly, the analysis focused on older reproductive-aged women, and therefore the results should not be generalized for younger women, as age might influence the microbial composition. Thirdly, the endometrial samples were obtained at different cycle phases, which restricts our ability to examine endometrial receptivity. Fourthly, despite taking utmost care to obtain fibroid-free tissue when sampling endometrial biopsies, the effect of fibroids on uterine microenvironment cannot be ruled out. Lastly, the study design lacked negative controls in the sampling process and separate validation, thus, stringent decontamination tools and strict data processing methods were applied.

In conclusion, our study results corroborate that the female upper reproductive tract harbours an endogenous microbiome, although with low microbial biomass. We observed that a significant portion of the microbial profile is shared between the FT and the endometrium, with approximately ~70% of the detected taxa being shared. Interestingly, women have unique microbial profiles, wherein two distinct tissues (FT and endometrium) displayed greater bacterial similarities than the same tissue sample (e.g. endometrium) between two individuals. Unravelling the female upper reproductive microbiome, helps understanding the natural microenvironment where crucial processes of oocyte fertilisation and embryo development occur. This knowledge can be used to improve *in vitro* fertilisation and embryo culture conditions for the treatment of infertility.

## Data availability statement

The data presented in the study are deposited in the NCBI SRA Database, accession number PRJNA915312 (Raw Data in Biom format).

## Ethics statement

The studies involving human participants were reviewed and approved by Ethics Research Committee (CEIC) of HCUVA, Murcia, Spain (Approval No. EST: 04/16). The patients/participants provided their written informed consent to participate in this study.

## Author contributions

AC-G, PC and SA conceived the idea and designed the study. PC, MP-S, MS-F provided all the required documentation to the Ethics Research Committee (CEIC) of Clinical University Hospital “Virgen de la Arrixaca” (HCUVA). MP-S, MS-F and AC-G recruited the patients. AC-G performed the sample and data collection. TE was the responsible of the registration, storage and processing of the samples at the Biobanco en Red de la Región de Murcia, BIOBANC-MUR. CR performed the amplification, library preparation and sequencing of the samples. IP-P, ES-E, IL-B analyzed the data. SA, AC-G and IP-P wrote the first manuscript draft. The manuscript was written through contributions of all authors. All authors have given approval to the final version of the manuscript. All authors contributed to the article and approved the submitted version.
