# Supplementary material for: The female upper reproductive tract harbors endogenous microbial profiles
Source: Front Endocrinol (Lausanne). 2023 Jun 21;14:1096050. doi: 10.3389/fendo.2023.1096050 (PMC10321600; doi:10.3389/fendo.2023.1096050)
Supplement: Supplementary file 1 [file DataSheet_1.pdf]

Supplementary Material

1 Supplementary Figures and Tables

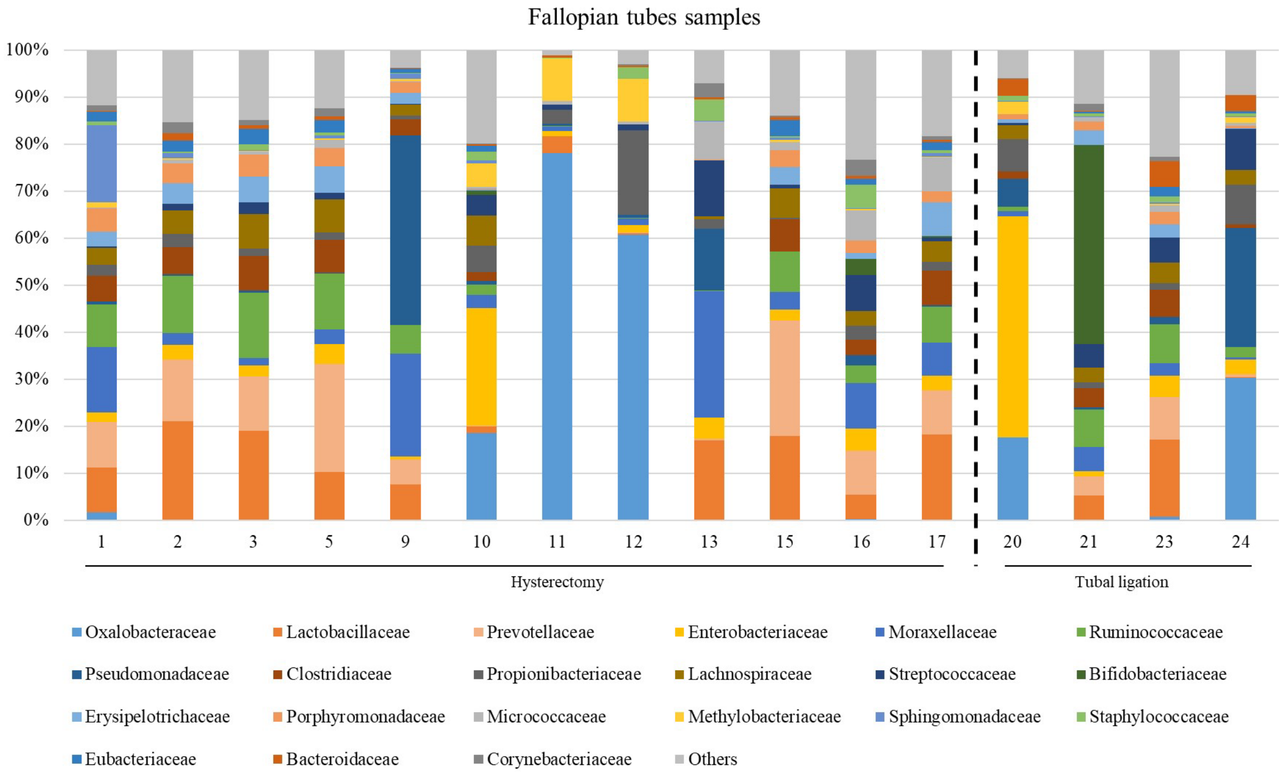

Supplementary Figure 1. The most prevalent families detected in the Fallopian tubes (FT) samples from patients underwent a total laparoscopic hysterectomy with bilateral salpingo-oophorectomy (patients 1, 2, 3, 5, 9, 10, 11, 12, 13, 15, 16 and 17) or laparoscopic tubal ligation (patients 20, 21, 23 and 24). Percent-stacked barchart of those families whose mean relative abundances were higher than 1% are represented.

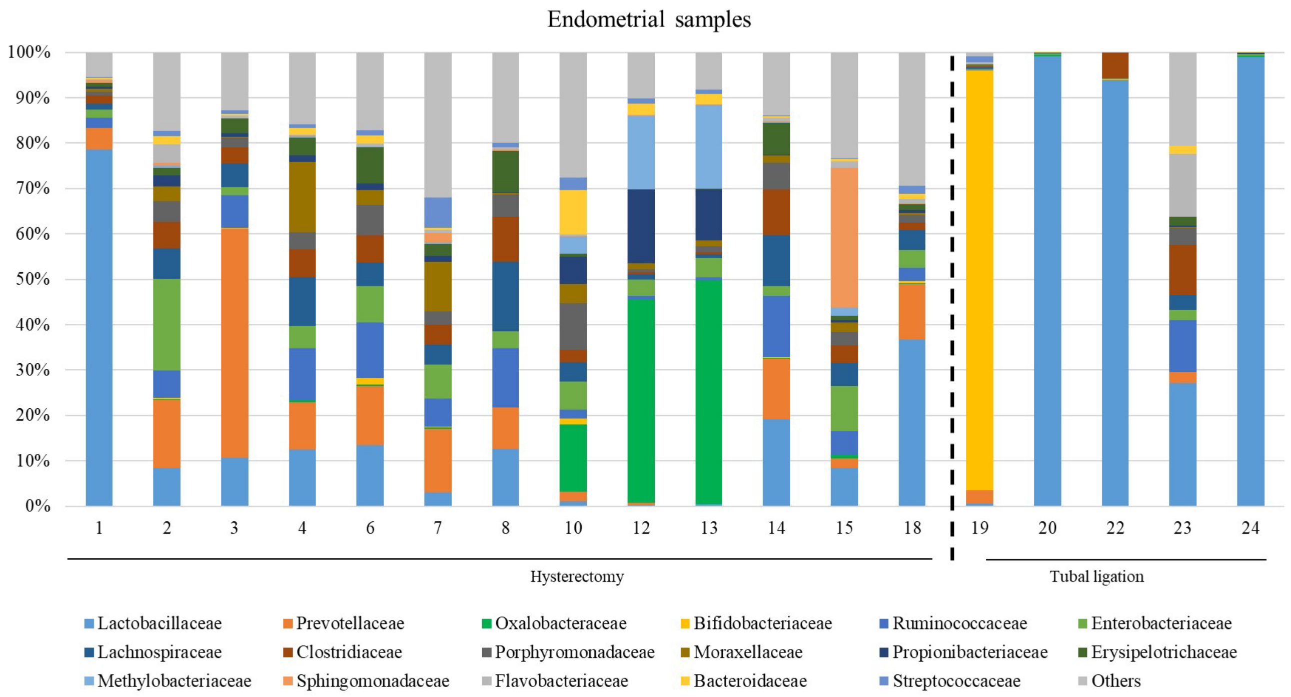

Supplementary Figure 2. The most prevalent families detected in the endometrial samples from patients undergoing a total laparoscopic hysterectomy with bilateral salpingo-oophorectomy (patients 1, 2, 3, 4, 6, 7, 8, 10, 12, 13, 14, 15, 18 and 19) or laparoscopic tubal ligation (patients 20, 22, 23 and 24). Percent-stacked barchart of those families whose mean relative abundances were higher than 1% are represented.

Supplementary Table 1. DNA concentration of the collected Fallopian tubes (FT) and endometrial (E) samples from hysterectomy (H) and tubal Ligation (TL) surgeries.

Patient	Surgery	Sample	Volume (µL)	Concentration [ng/µl]	A260/A280
1	H	FT	80	304,3	1,85
		E	140	146,3	1,8
2	H	FT	95	21,2	1,7
		E	85	351,6	1,81
3	H	FT	90	197,6	1,83
		E	90	739,9	1,81
4	H	FT	95	616,4	1,76
5	H	FT	115	42,2	1,8
6	H	FT	95	19,8	1,61
7	H	FT	95	396,1	1,8
8	H	FT	60	964,3	1,87
9	H	FT	70	623,5	1,87
10	H	FT	75	3494,4	1,87
		E	55	2964	1,81
11	H	FT	37	4302	2,01
12	H	FT	79	881,9	2,14
		E	84	1368,6	1,98
13	H	FT	76	5119,4	1,93
		E	65	4559,4	1,99
14	H	FT	95	686,4	1,91
15	H	FT	96	18,7	1,71
		E	95	771,2	1,6
16	H	FT	80	1097,3	1,87
17	H	FT	100	48,1	1,74
18	H	FT	100	50,3	1,8
19	H	FT	100	704,9	1,75
20	TL	FT	97	5872,9	1,94
		E	76	2098,2	1,76
21	TL	FT	75	1976,3	1,86
22	TL	FT	70	367,7	1,59
23	TL	FT	90	93,1	1,84
		E	75	1318,1	1,77
24	TL	FT	77	1334,8	2,12
		E	78	1874,1	1,72

Supplementary Table 2. Sets of primers, which targeted the regions V2, V4, and V8, and V3, V6-7, and V9, correspondingly used to amplify the 16S rRNA gene by multiplex PCR using Ion Torrent 16S Metagenomics kit (Ion Torrent, Thermo Fisher Scientific Inc, USA).

Primer	Sequence 5´ to 3´	PCR size product (bp)
V2_F	GGCGSACGGGTGAGTAA	250 pb
V2_R	GCTGCCTCCCGTAGGAGT	
V3_F	ACTGAGACACGGTCCARACT	215 pb
V3_R	GTATTACCGCGGCTGCTG	
V4_F	CCAGCAGCCGCGGTAATA	288 pb
V4_R	GGACTACCAGGGTATCTAATCCTGT	
V6-7_F	ACAAGCGGHGGARCATGT	260 pb
V6-7_R	GACGTCATCCCCACCTTCC	
V8_F	GYTGTCGTCAGCTCGTGT	295 pb
V8_R	CGATTACTAGCGAYTCCGACTTCA	
V9_F	GTTACGACTTCACCCCAGTCA	209 pb
V9_R	GCGTCGTAGTCCGGATTGG	

Supplementary Table 3. Demographic characteristics and collected samples for each patient that participated in the study. The 24 participants were also asked to report their age, body mass index (BMI) and obstetric formula [G: gravida (number of pregnancies); P: parity (number of births of viable offspring); A: abortus (abortions)]. UF (Uterine Fibroids), C (healthy women that desires definitive contraceptive method), H (Laparoscopic hysterectomy), TL (Laparoscopic Tubal ligation). The uterine cycle phase (Proliferative or Secretory) was calculated according to their menstrual cycle and confirmed after the histological study. The samples corresponding to unclear results from histological study were classified as “Unknown”.

Patient	Pathology	Surgery	Age	BMI	Uterine cycle phase	Obstetric history	Collected Samples	
							Fallopian tubes	Endometrium
1	UF	H	42	35	Unknown	G2P2	1	1
2	UF	H	42	28	Secretory	G1P1	1	1
3	UF	H	44	30	Proliferative	G1P1	1	1
4	UF	H	48	26,5	Proliferative	G1P1	0	1
5	UF	H	46	23	Secretory	G2P2	1	0
6	UF	H	45	35	Secretory	G3P2A1	0	1
7	UF	H	41	32,1	Proliferative	G1P1	0	1
8	UF	H	49	27,9	Unknown	G3P3	0	1
9	UF	H	50	30,3	Secretory	G1P1	1	0
10	UF	H	43	27,4	Proliferative	G2P2	1	1
11	UF	H	43	20,2	Secretory	G4P3A1	1	0
12	UF	H	43	23,8	Proliferative	G2P2	1	1

13	UF	H	46	24,1	Secretory	G1P1	1	1
14	UF	H	43	32,9	Secretory	G4P3A1	0	1
15	UF	H	47	28,1	Proliferative	G1P1	1	1
16	UF	H	40	32,4	Unknown	G1P1	1	0
17	UF	H	43	20,2	Secretory	G2P2	1	0
18	UF	H	48	34,5	Proliferative	G4P4	0	1
19	UF	H	48	30,4	Proliferative	G2P2	0	1
20	C	TL	33	25,7	Proliferative	G3P3	1	1
21	C	TL	39	26	Secretory	G8P2A6	1	0
22	C	TL	41	24,6	Unknown	G2P2	0	1
23	C	TL	40	32,1	Unknown	G2P2	1	1
24	C	TL	31	33,1	Secretory	G3P2A1	1	1

Supplementary Table 4. List of bacteria genera (n=77) identified in Fallopian tubes samples (n=16) after applying the decontamination method Decontam and an additional filtering step to eliminate those bacterial taxa present in less than 30% of the participants as previously described (24). Relative abundances of detected genera are expressed as median and first and third quartiles (q1, q3) and were analyzed by non-parametric Mann-Whitney *U* test. P-values were adjusted using the method of Benjamini and Hochberg to control for the multiple testing (false discovery rate, FDR).

	**Fallopian Tubes (n=16)**	
n	Bacteria Genera	Median [q1; q3]
1	*Lactobacillus*	14.3 [3.48;24.4]
2	*Prevotella*	9.29 [0.31;12.7]
3	*Acinetobacter*	3.20 [1.36;11.7]
4	*Propionibacterium*	3.09 [2.45;5.86]
5	*Faecalibacterium*	3.09 [0.68;4.97]
6	*Clostridium*	2.15 [0.74;4.11]
7	*Streptococcus*	2.08 [1.02;8.16]
8	*Catenibacterium*	2.03 [0.00;3.57]
9	*Staphylococcus*	1.45 [0.62;2.67]
10	*Pseudomonas*	1.20 [0.59;6.56]
11	*Corynebacterium*	1.12 [0.27;2.85]
12	*Ruminococcus2*	0.89 [0.11;1.51]
13	*Dorea*	0.88 [0.57;1.53]
14	*Ruminococcus*	0.75 [0.14;1.53]
15	*Gemmiger*	0.61 [0.23;0.79]
16	*Methylobacterium*	0.57 [0.28;3.06]
17	*Bacteroides*	0.56 [0.14;0.97]
18	*Eubacterium2*	0.52 [0.03;1.97]
19	*Parabacteroides*	0.49 [0.00;0.74]
20	*Eubacterium*	0.40 [0.11;1.03]
21	*Arthrobacter*	0.36 [0.20;0.88]
22	*Blautia*	0.31 [0.08;1.03]

23	*Dolosigranulum*	0.27 [0.00;0.73]
24	*Roseburia*	0.23 [0.09;0.39]
25	*Massilia*	0.22 [0.04;23.9]
26	*Enterococcus*	0.19 [0.00;0.78]
27	*Sphingomonas*	0.17 [0.00;0.57]
28	*Pelomonas*	0.16 [0.00;0.58]
29	*Collinsella*	0.16 [0.00;0.40]
30	*Kocuria*	0.15 [0.03;0.24]
31	*Micrococcus*	0.15 [0.00;0.41]
32	*Microbacterium*	0.13 [0.00;0.49]
33	*Coprococcus*	0.09 [0.00;0.41]
34	*Ralstonia*	0.08 [0.00;0.28]
35	*Ruminiclostridium*	0.07 [0.00;0.22]
36	*Neisseria*	0.07 [0.00;0.21]
37	*Enhydrobacter*	0.06 [0.00;0.28]
38	*Granulicatella*	0.06 [0.00;0.17]
39	*Anaerococcus*	0.05 [0.00;0.23]
40	*Lachnoclostridium*	0.05 [0.00;0.10]
41	*Gemella*	0.04 [0.00;0.26]
42	*Rothia*	0.04 [0.00;0.16]
43	*Cloacibacterium*	0.03 [0.00;1.10]
44	*Bilophila*	0.03 [0.00;0.21]
45	*Subdoligranulum*	0.03 [0.00;0.15]
46	*Lactococcus*	0.03 [0.00;0.10]
47	*Campylobacter*	0.02 [0.00;0.23]
48	*Dialister*	0.02 [0.00;0.22]
49	*Rhizobium*	0.02 [0.00;0.20]
50	*Haemophilus*	0.02 [0.00;0.17]
51	*Moraxella*	0.02 [0.00;0.11]
52	*Megasphaera*	0.01 [0.00;0.11]
53	*Oxalicibacterium*	0.00 [0.00;6.06]
54	*Paracoccus*	0.00 [0.00;0.43]
55	*Helicobacter*	0.00 [0.00;0.31]
56	*Bacillus*	0.00 [0.00;0.28]
57	*Brevundimonas*	0.00 [0.00;0.26]
58	*Pseudoflavonifractor*	0.00 [0.00;0.20]
59	*Alistipes*	0.00 [0.00;0.13]
60	*Phascolarctobacterium*	0.00 [0.00;0.12]
61	*Fusobacterium*	0.00 [0.00;0.10]
62	*Bifidobacterium*	0.00 [0.00;0.09]
63	*Flavonifractor*	0.00 [0.00;0.09]
64	*Desulfovibrio*	0.00 [0.00;0.09]
65	*Butyricimonas*	0.00 [0.00;0.08]
66	*Porphyromonas*	0.00 [0.00;0.08]
67	*Aeromonas*	0.00 [0.00;0.07]

68	*Finegoldia*	0.00 [0.00;0.07]
69	*Brachyspira*	0.00 [0.00;0.06]
70	*Burkholderia*	0.00 [0.00;0.06]
71	*Comamonas*	0.00 [0.00;0.06]
72	*Shewanella*	0.00 [0.00;0.06]
73	*Sutterella*	0.00 [0.00;0.05]
74	*Actinomyces*	0.00 [0.00;0.05]
75	*Parasutterella*	0.00 [0.00;0.04]
76	*Herbaspirillum*	0.00 [0.00;0.04]
77	*Mitsuokella*	0.00 [0.00;0.04]

Supplementary Table 5. List of bacteria genera (n=70) identified in endometrial samples (n=18) after applying the decontamination method Decontam and an additional filtering step to eliminate those bacterial taxa present in less than 30% of the participants as previously described (24). Relative abundances of detected genera are expressed as median and first and third quartiles (q1, q3) and were analyzed by non-parametric Mann-Whitney *U* test. P-values were adjusted using the method of Benjamini and Hochberg to control for the multiple testing (false discovery rate, FDR).

	**Endometrium (n=18)**	
n	Bacteria Genera	Median [q1; q3]
1	*Lactobacillus*	23.0 [6.89;49.8]
2	*Prevotella*	4.13 [0.85;13.7]
3	*Faecalibacterium*	2.18 [0.24;4.12]
4	*Clostridium*	2.08 [0.32;5.06]
5	*Streptococcus*	1.01 [0.18;2.07]
6	*Propionibacterium*	0.94 [0.31;2.67]
7	*Acinetobacter*	0.77 [0.12;3.18]
8	*Staphylococcus*	0.75 [0.09;1.34]
9	*Bacteroides*	0.62 [0.09;1.13]
10	*Eubacterium*	0.50 [0.00;0.89]
11	*Gemmiger*	0.47 [0.00;0.98]
12	*Dorea*	0.42 [0.09;1.05]
13	*Blautia*	0.38 [0.06;0.70]
14	*Ruminococcus*	0.36 [0.21;1.10]
15	*Roseburia*	0.34 [0.00;0.71]
16	*Ruminococcus2*	0.33 [0.03;1.02]
17	*Arthrobacter*	0.31 [0.09;1.13]
18	*Corynebacterium*	0.31 [0.06;0.91]
19	*Catenibacterium*	0.31 [0.00;2.22]
20	*Methylobacterium*	0.22 [0.04;0.63]
21	*Eubacterium2*	0.20 [0.00;1.22]
22	*Parabacteroides*	0.18 [0.00;0.77]
23	*Phascolarctobacterium*	0.16 [0.00;0.47]
24	*Coprococcus*	0.13 [0.00;0.38]
25	*Pseudomonas*	0.11 [0.01;0.51]

26	*Bilophila*	0.11 [0.00;0.51]
27	*Cloacibacterium*	0.11 [0.00;0.45]
28	*Massilia*	0.10 [0.00;0.20]
29	*Microbacterium*	0.09 [0.00;0.18]
30	*Collinsella*	0.08 [0.00;0.25]
31	*Subdoligranulum*	0.07 [0.00;0.34]
32	*Moraxella*	0.06 [0.00;0.17]
33	*Enterococcus*	0.05 [0.00;0.55]
34	*Pelomonas*	0.05 [0.00;0.35]
35	*Dialister*	0.04 [0.00;0.08]
36	*Ralstonia*	0.03 [0.00;0.23]
37	*Campylobacter*	0.03 [0.00;0.20]
38	*Dolosigranulum*	0.00 [0.00;0.34]
39	*Gardnerella*	0.00 [0.00;0.29]
40	*Butyricimonas*	0.00 [0.00;0.29]
41	*Herbaspirillum*	0.00 [0.00;0.23]
42	*Desulfovibrio*	0.00 [0.00;0.20]
43	*Helicobacter*	0.00 [0.00;0.17]
44	*Finegoldia*	0.00 [0.00;0.16]
45	*Anaerococcus*	0.00 [0.00;0.13]
46	*Veillonella*	0.00 [0.00;0.12]
47	*Megasphaera*	0.00 [0.00;0.12]
48	*Mitsuokella*	0.00 [0.00;0.12]
49	*Kocuria*	0.00 [0.00;0.11]
50	*Lactococcus*	0.00 [0.00;0.10]
51	*Pseudoflavonifractor*	0.00 [0.00;0.10]
52	*Oxalicibacterium*	0.00 [0.00;0.09]
53	*Sphingomonas*	0.00 [0.00;0.08]
54	*Oscillibacter*	0.00 [0.00;0.07]
55	*Vibrio*	0.00 [0.00;0.07]
56	*Actinomyces*	0.00 [0.00;0.07]
57	*Micrococcus*	0.00 [0.00;0.07]
58	*Barnesiella*	0.00 [0.00;0.06]
59	*Olsenella*	0.00 [0.00;0.06]
60	*Neisseria*	0.00 [0.00;0.06]
61	*Ruminiclostridium*	0.00 [0.00;0.06]
62	*Klebsiella*	0.00 [0.00;0.05]
63	*Gemella*	0.00 [0.00;0.05]
64	*Brachymonas*	0.00 [0.00;0.04]
65	*Serratia*	0.00 [0.00;0.04]
66	*Rothia*	0.00 [0.00;0.04]
67	*Lachnoclostridium*	0.00 [0.00;0.03]
68	*Porphyromonas*	0.00 [0.00;0.03]
69	*Chryseobacterium*	0.00 [0.00;0.02]
70	*Bacillus*	0.00 [0.00;0.02]

Supplementary Table 6. Fallopian tubes microbial taxa comparisons between fertile women with benign uterine condition and fertile women without the disease, i.e. sampling methods (hysterectomy *vs.* tubal ligation). Relative abundances of detected genera are expressed as median and first and third quartiles (q1, q3) and were analyzed by non-parametric Mann-Whitney *U* test. P-values were adjusted using the method of Benjamini and Hochberg to control for the multiple testing (false discovery rate, FDR).

	**Tubal ligation (n=4)**	**Hysterectomy (n=12)**		
	Median [q; q3]	Median [q; q3]	P-value	Adjusted P-value
Acinetobacter	2.59 [1.60;5.39]	3.95 [1.36;13.3]	0.716	0.934
Actinomyces	0.05 [0.00;0.13]	0.00 [0.00;0.01]	0.376	0.934
Aeromonas	0.00 [0.00;0.00]	0.00 [0.00;0.09]	0.140	0.934
Alistipes	0.06 [0.00;0.15]	0.00 [0.00;0.09]	0.789	0.940
Anaerococcus	0.22 [0.15;0.31]	0.02 [0.00;0.17]	0.255	0.934
Arthrobacter	0.36 [0.27;0.46]	0.39 [0.20;1.32]	0.715	0.934
Bacillus	0.15 [0.00;0.49]	0.00 [0.00;0.13]	0.486	0.934
Bacteroides	2.92 [2.18;3.03]	0.50 [0.14;0.63]	0.114	0.934
Bifidobacterium	0.00 [0.00;0.03]	0.00 [0.00;0.09]	0.626	0.934
Bilophila	0.60 [0.45;0.74]	0.00 [0.00;0.13]	0.045	0.934
Blautia	0.60 [0.12;1.07]	0.31 [0.08;0.64]	0.808	0.940
Brachyspira	0.00 [0.00;0.11]	0.00 [0.00;0.06]	1.000	1.000
Brevundimonas	0.18 [0.00;0.44]	0.00 [0.00;0.06]	0.461	0.934
Burkholderia	0.00 [0.00;0.01]	0.00 [0.00;0.06]	0.365	0.934
Butyricimonas	0.03 [0.00;0.18]	0.00 [0.00;0.04]	0.376	0.934
Campylobacter	0.19 [0.09;0.32]	0.00 [0.00;0.11]	0.218	0.934
Catenibacterium	1.04 [0.00;2.81]	2.11 [0.00;3.57]	0.709	0.934
Cloacibacterium	0.12 [0.00;0.41]	0.03 [0.00;1.64]	0.796	0.940
Clostridium	2.90 (2.86)	2.43 (1.89)	0.773	0.940
Collinsella	0.28 [0.18;0.41]	0.09 [0.00;0.40]	0.575	0.934
Comamonas	0.00 [0.00;0.00]	0.00 [0.00;0.07]	0.140	0.934
Coprococcus	0.13 [0.00;0.29]	0.09 [0.00;0.52]	0.704	0.934
Corynebacterium	0.81 [0.27;2.03]	1.42 [0.27;2.85]	0.903	0.940
Desulfovibrio	0.03 [0.00;0.23]	0.00 [0.00;0.09]	0.789	0.940
Dialister	0.02 [0.00;0.10]	0.03 [0.00;0.22]	0.897	0.940
Dolosigranulum	0.19 [0.14;0.27]	0.51 [0.00;0.80]	0.460	0.934
Dorea	1.33 (1.16)	1.04 (0.82)	0.666	0.934
Enhydrobacter	0.04 [0.00;0.20]	0.07 [0.00;0.28]	0.704	0.934
Enterococcus	0.51 [0.09;1.63]	0.13 [0.00;0.60]	0.376	0.934
Eubacterium	0.42 (0.42)	0.69 (0.65)	0.359	0.934
Eubacterium2	0.62 [0.03;1.24]	0.52 [0.21;2.32]	0.502	0.934
Faecalibacterium	3.17 (3.38)	3.17 (2.54)	1.000	1.000
Finegoldia	0.03 [0.00;0.83]	0.00 [0.00;0.07]	0.486	0.934
Flavonifractor	0.05 [0.00;0.11]	0.00 [0.00;0.02]	0.461	0.934

Fusobacterium	0.00 [0.00;0.15]	0.00 [0.00;0.10]	0.883	0.940
Gemella	0.47 [0.15;0.87]	0.00 [0.00;0.08]	0.136	0.934
Gemmiger	0.72 (0.89)	0.61 (0.48)	0.824	0.940
Granulicatella	0.62 [0.04;1.42]	0.04 [0.00;0.15]	0.255	0.934
Haemophilus	0.07 [0.00;2.19]	0.02 [0.00;0.17]	0.796	0.940
Helicobacter	0.00 [0.00;0.09]	0.00 [0.00;0.35]	0.531	0.934
Herbaspirillum	0.04 [0.00;0.13]	0.00 [0.00;0.01]	0.238	0.934
Kocuria	0.07 [0.00;0.17]	0.15 [0.06;0.35]	0.328	0.934
Lachnoclostridium	0.00 [0.00;0.01]	0.05 [0.00;0.12]	0.164	0.934
Lactobacillus	9.13 (11.4)	16.4 (11.7)	0.318	0.934
Lactococcus	0.03 [0.00;0.07]	0.03 [0.00;0.16]	0.698	0.934
Massilia	11.4 [0.37;25.5]	0.11 [0.02;8.48]	0.181	0.934
Megasphaera	0.00 [0.00;0.04]	0.04 [0.00;0.13]	0.331	0.934
Methylobacterium	0.85 [0.22;2.76]	0.57 [0.37;3.72]	0.671	0.934
Microbacterium	0.11 [0.00;0.25]	0.20 [0.00;0.71]	0.376	0.934
Micrococcus	0.46 [0.00;1.04]	0.15 [0.05;0.23]	0.902	0.940
Mitsuokella	0.00 [0.00;0.01]	0.00 [0.00;0.04]	0.768	0.940
Moraxella	0.06 [0.00;0.14]	0.02 [0.00;0.10]	0.698	0.934
Neisseria	0.40 [0.27;0.60]	0.06 [0.00;0.11]	0.085	0.934
Oxalicibacterium	5.03 [0.00;10.2]	0.00 [0.00;1.18]	0.461	0.934
Parabacteroides	0.15 [0.08;0.32]	0.60 [0.00;1.06]	0.460	0.934
Paracoccus	0.34 [0.00;0.74]	0.00 [0.00;0.19]	0.593	0.934
Parasutterella	0.00 [0.00;0.01]	0.00 [0.00;0.05]	0.555	0.934
Pelomonas	0.18 [0.00;0.40]	0.16 [0.00;0.87]	0.704	0.934
Phascolarctobacterium	0.05 [0.00;0.12]	0.00 [0.00;0.12]	0.894	0.940
Porphyromonas	0.05 [0.00;0.12]	0.00 [0.00;0.02]	0.302	0.934
Prevotella	4.37 [0.27;9.70]	10.4 [0.31;13.3]	0.504	0.934
Propionibacterium	6.24 [2.67;11.6]	3.09 [2.45;4.41]	0.628	0.934
Pseudoflavonifractor	0.02 [0.00;0.09]	0.00 [0.00;0.20]	0.894	0.940
Pseudomonas	7.42 [3.10;15.8]	0.82 [0.51;2.21]	0.069	0.934
Ralstonia	0.05 [0.00;0.13]	0.11 [0.02;0.34]	0.389	0.934
Rhizobium	0.28 [0.15;0.63]	0.00 [0.00;0.12]	0.060	0.934
Roseburia	0.27 [0.22;0.35]	0.19 [0.08;0.39]	0.544	0.934
Rothia	0.00 [0.00;0.11]	0.09 [0.00;0.16]	0.560	0.934
Ruminiclostridium	0.05 [0.00;0.11]	0.11 [0.00;0.35]	0.376	0.934
Ruminococcus	0.35 [0.00;0.75]	0.85 [0.33;2.17]	0.179	0.934
Ruminococcus2	0.89 [0.66;1.05]	0.85 [0.10;1.69]	0.903	0.940
Shewanella	0.00 [0.00;0.00]	0.00 [0.00;0.08]	0.140	0.934
Sphingomonas	0.05 [0.00;0.15]	0.20 [0.10;0.83]	0.176	0.934
Staphylococcus	1.59 [1.34;1.87]	1.22 [0.57;2.79]	0.716	0.934
Streptococcus	8.67 [6.00;10.5]	1.61 [0.91;3.47]	0.182	0.934
Subdoligranulum	0.06 [0.00;0.13]	0.03 [0.00;0.15]	1.000	1.000
Sutterella	0.03 [0.00;0.08]	0.00 [0.00;0.05]	0.486	0.934

Supplementary Table 7. Endometrial microbial taxa comparisons between fertile women with benign uterine condition and fertile women without the disease, i.e. sampling methods (hysterectomy *vs.* tubal ligation). Relative abundances of detected genera are expressed as median and first and third quartiles (q1, q3) and were analyzed by non-parametric Mann-Whitney *U* test. P-values were adjusted using the method of Benjamini and Hochberg to control for the multiple testing (false discovery rate, FDR).

	**Tubal ligation (n=4)**	**Hysterectomy (n=14)**		
	Median [q1; q3]	Median [q1; q3]	P-value	Adjusted P-value
Acinetobacter	**0.02 [0.00;0.09]**	**1.31 [0.42;4.70]**	**0.011**	**0.083**
Actinomyces	0.00 [0.00;0.01]	0.00 [0.00;0.08]	0.487	0.541
Anaerococcus	0.00 [0.00;0.00]	0.05 [0.00;0.13]	0.062	0.181
Arthrobacter	**0.00 [0.00;0.03]**	**0.56 [0.27;1.43]**	**0.006**	**0.083**
Bacillus	0.00 [0.00;0.00]	0.00 [0.00;0.04]	0.179	0.232
Bacteroides	0.07 [0.04;0.80]	0.72 [0.24;1.13]	0.203	0.258
Barnesiella	0.00 [0.00;0.18]	0.00 [0.00;0.06]	0.950	0.950
Bilophila	0.00 [0.00;0.10]	0.17 [0.01;0.59]	0.155	0.232
Blautia	**0.00 [0.00;0.13]**	**0.45 [0.15;1.06]**	**0.048**	0.161
Brachymonas	0.00 [0.00;0.00]	0.00 [0.00;0.06]	0.179	0.232
Butyricimonas	0.00 [0.00;0.34]	0.03 [0.00;0.29]	0.600	0.636
Campylobacter	0.00 [0.00;0.02]	0.08 [0.00;0.23]	0.140	0.228
Catenibacterium	0.00 [0.00;0.07]	0.80 [0.16;2.99]	0.035	0.153
Chryseobacterium	0.00 [0.00;0.00]	0.00 [0.00;0.04]	0.179	0.232
Cloacibacterium	**0.00 [0.00;0.00]**	**0.15 [0.02;0.59]**	**0.026**	0.123
Clostridium	1.43 [0.00;5.65]	2.09 [0.56;5.06]	0.523	0.563
Collinsella	0.00 [0.00;0.05]	0.10 [0.00;0.33]	0.165	0.232
Coprococcus	**0.00 [0.00;0.00]**	**0.22 [0.04;0.39]**	**0.009**	**0.083**
Corynebacterium	**0.00 [0.00;0.12]**	**0.47 [0.11;2.23]**	**0.025**	0.123
Desulfovibrio	0.00 [0.00;0.14]	0.06 [0.00;0.20]	0.521	0.563
Dialister	**0.00 [0.00;0.00]**	**0.07 [0.01;0.10]**	**0.026**	0.123
Dolosigranulum	0.00 [0.00;0.00]	0.09 [0.00;0.46]	0.091	0.193
Dorea	**0.00 [0.00;0.20]**	**0.70 [0.18;1.24]**	**0.037**	0.154
Enterococcus	**0.00 [0.00;0.00]**	**0.15 [0.01;0.63]**	**0.026**	0.123
Eubacterium	0.00 [0.00;0.23]	0.55 [0.20;1.41]	0.144	0.229
Eubacterium2	**0.00 [0.00;0.05]**	**0.74 [0.06;1.38]**	**0.045**	0.159
Faecalibacterium	**0.00 [0.00;0.90]**	**3.16 [0.79;4.32]**	**0.043**	0.159
Finegoldia	0.00 [0.00;0.00]	0.03 [0.00;0.20]	0.091	0.193
Gardnerella	0.00 [0.00;0.02]	0.01 [0.00;0.51]	0.268	0.329
Gemella	0.00 [0.00;0.00]	0.00 [0.00;0.07]	0.179	0.232
Gemmiger	0.00 [0.00;1.25]	0.56 [0.15;0.98]	0.304	0.361
Helicobacter	0.00 [0.00;0.00]	0.00 [0.00;0.30]	0.129	0.215
Herbaspirillum	0.00 [0.00;0.00]	0.02 [0.00;0.26]	0.091	0.193
Klebsiella	0.00 [0.00;0.00]	0.00 [0.00;0.07]	0.129	0.215
Kocuria	0.00 [0.00;0.00]	0.06 [0.00;0.14]	0.062	0.181

Lachnoclostridium	0.00 [0.00;0.05]	0.00 [0.00;0.03]	0.893	0.906
Lactobacillus	**98.2 [85.5;99.4]**	**18.7 [2.31;24.8]**	**0.004**	**0.083**
Lactococcus	0.00 [0.00;0.00]	0.03 [0.00;0.12]	0.091	0.193
Massilia	0.06 [0.00;0.13]	0.11 [0.00;0.25]	0.437	0.493
Megasphaera	0.00 [0.00;0.00]	0.02 [0.00;0.19]	0.091	0.193
Methylobacterium	**0.00 [0.00;0.01]**	**0.33 [0.13;3.84]**	**0.006**	**0.083**
Microbacterium	**0.00 [0.00;0.00]**	**0.14 [0.02;0.39]**	**0.026**	0.123
Micrococcus	0.00 [0.00;0.00]	0.01 [0.00;0.15]	0.091	0.193
Mitsuokella	0.00 [0.00;0.01]	0.02 [0.00;0.14]	0.268	0.329
Moraxella	0.00 [0.00;0.02]	0.10 [0.00;0.32]	0.107	0.208
Neisseria	0.00 [0.00;0.00]	0.00 [0.00;0.10]	0.129	0.215
Olsenella	0.00 [0.00;0.00]	0.00 [0.00;0.07]	0.129	0.215
Oscillibacter	0.00 [0.00;0.06]	0.00 [0.00;0.07]	0.809	0.833
Oxalicibacterium	0.07 [0.04;0.10]	0.00 [0.00;0.08]	0.382	0.438
Parabacteroides	0.00 [0.00;0.07]	0.30 [0.01;0.97]	0.101	0.202
Pelomonas	0.00 [0.00;0.01]	0.14 [0.00;0.40]	0.085	0.193
Phascolarctobacterium	0.00 [0.00;2.41]	0.23 [0.08;0.47]	0.304	0.361
Porphyromonas	0.00 [0.00;0.00]	0.00 [0.00;0.08]	0.179	0.232
Prevotella	**0.00 [0.00;0.44]**	**12.3 [3.12;15.8]**	**0.009**	**0.083**
Propionibacterium	**0.20 [0.09;0.32]**	**1.76 [0.64;4.45]**	**0.015**	0.102
Pseudoflavonifractor	0.00 [0.00;0.14]	0.00 [0.00;0.10]	0.717	0.749
Pseudomonas	0.00 [0.00;0.12]	0.18 [0.06;0.80]	0.086	0.193
Ralstonia	**0.00 [0.00;0.00]**	**0.12 [0.00;0.25]**	**0.041**	0.159
Roseburia	**0.00 [0.00;0.00]**	**0.56 [0.19;0.99]**	**0.009**	**0.083**
Rothia	0.00 [0.00;0.00]	0.00 [0.00;0.06]	0.179	0.232
Ruminiclostridium	0.00 [0.00;0.00]	0.00 [0.00;0.06]	0.129	0.215
Ruminococcus	0.00 [0.00;0.45]	0.40 [0.26;1.10]	0.098	0.202
Ruminococcus2	0.00 [0.00;0.10]	0.47 [0.16;1.44]	0.053	0.170
Serratia	0.00 [0.00;0.00]	0.00 [0.00;0.09]	0.179	0.232
Sphingomonas	0.00 [0.00;0.00]	0.03 [0.00;0.23]	0.091	0.193
Staphylococcus	**0.00 [0.00;0.01]**	**1.04 [0.51;1.56]**	**0.003**	**0.083**
Streptococcus	**0.00 [0.00;0.07]**	**1.18 [0.53;2.23]**	**0.006**	**0.083**
Subdoligranulum	0.00 [0.00;0.15]	0.10 [0.01;0.34]	0.381	0.438
Veillonella	0.00 [0.00;0.00]	0.00 [0.00;0.17]	0.129	0.215
Vibrio	0.00 [0.00;0.00]	0.00 [0.00;0.09]	0.179	0.232

Supplementary Table 8. Differentially abundant microbial taxa between E and FT samples. Relative abundances of detected genera are expressed as median and first and third quartiles (q1, q3) and were analyzed by non-parametric Mann-Whitney *U* test and ANCOM-BC. P-values were adjusted using the method of Benjamini and Hochberg to control for the multiple testing (false discovery rate, FDR).

	**Fallopian Tubes (n=16)**	**Endometrium (n=14)**	**Mann-Whitney**	**ANCOM-BC**

	Median [q1; q3]	Median [q1; q3]	P-value	Adjusted P-value	P-value	Adjusted P-value
Acinetobacter	3.20 [1.36;11.7]	1.31 [0.42;4.70]	0.135	0.344	0,136	0,370
Actinomyces	0.00 [0.00;0.05]	0.00 [0.00;0.08]	0.747	0.938	0,754	0,926
Aeromonas	**0.00 [0.00;0.07]**	**0.00 [0.00;0.00]**	**0.025**	**0.075**	**0,000**	**0,000**
Alistipes	**0.00 [0.00;0.13]**	**0.00 [0.00;0.00]**	**0.006**	0.048	**0,000**	**0,000**
Anaerococcus	0.05 [0.00;0.23]	0.05 [0.00;0.13]	0.761	0.938	0,864	0,960
Arthrobacter	0.36 [0.20;0.88]	0.56 [0.27;1.43]	0.429	0.674	0,140	0,370
Bacillus	0.00 [0.00;0.28]	0.00 [0.00;0.04]	0.470	0.706	0,314	0,530
Bacteroides	0.56 [0.14;0.97]	0.72 [0.24;1.13]	0.533	0.772	0,217	0,454
Barnesiella	**0.00 [0.00;0.00]**	**0.00 [0.00;0.06]**	**0.010**	**0.053**	**0,000**	**0,000**
Bifidobacterium	**0.00 [0.00;0.09]**	**0.00 [0.00;0.00]**	**0.012**	**0.054**	**0,000**	**0,000**
Bilophila	0.03 [0.00;0.21]	0.17 [0.01;0.59]	0.246	0.510	0,234	0,462
Blautia	0.31 [0.08;1.03]	0.45 [0.15;1.06]	0.441	0.674	0,293	0,521
Brachymonas	**0.00 [0.00;0.00]**	**0.00 [0.00;0.06]**	**0.010**	**0.053**	**0,000**	**0,000**
Brachyspira	**0.00 [0.00;0.06]**	**0.00 [0.00;0.00]**	**0.025**	**0.075**	**0,000**	**0,000**
Brevundimonas	**0.00 [0.00;0.26]**	**0.00 [0.00;0.00]**	**0.025**	**0.075**	**0,000**	**0,000**
Burkholderia	**0.00 [0.00;0.06]**	**0.00 [0.00;0.00]**	**0.012**	**0.054**	**0,000**	**0,000**
Butyricimonas	0.00 [0.00;0.08]	0.03 [0.00;0.29]	0.336	0.590	0,345	0,566
Campylobacter	0.02 [0.00;0.23]	0.08 [0.00;0.23]	0.599	0.801	0,586	0,772
Catenibacterium	2.03 [0.00;3.57]	0.80 [0.16;2.99]	0.899	0.967	0,632	0,809
Chryseobacterium	**0.00 [0.00;0.00]**	**0.00 [0.00;0.04]**	**0.010**	**0.053**	**0,000**	**0,000**
Cloacibacterium	0.03 [0.00;1.10]	0.15 [0.02;0.59]	0.667	0.880	0,463	0,683
Clostridium	2.15 [0.74;4.11]	2.09 [0.56;5.06]	0.835	0.967	0,553	0,740
Collinsella	0.16 [0.00;0.40]	0.10 [0.00;0.33]	0.898	0.967	0,983	0,991
Comamonas	**0.00 [0.00;0.06]**	**0.00 [0.00;0.00]**	**0.025**	**0.075**	**0,000**	**0,000**
Coprococcus	0.09 [0.00;0.41]	0.22 [0.04;0.39]	0.423	0.674	0,232	0,462
Corynebacterium	1.12 [0.27;2.85]	0.47 [0.11;2.23]	0.561	0.774	0,991	0,991
Desulfovibrio	0.00 [0.00;0.09]	0.06 [0.00;0.20]	0.430	0.674	0,529	0,730
Dialister	0.02 [0.00;0.22]	0.07 [0.01;0.10]	0.414	0.674	0,391	0,607
Dolosigranulum	0.27 [0.00;0.73]	0.09 [0.00;0.46]	0.334	0.590	0,293	0,521
Dorea	0.88 [0.57;1.53]	0.70 [0.18;1.24]	0.546	0.774	0,778	0,926
Enhydrobacter	**0.06 [0.00;0.28]**	**0.00 [0.00;0.00]**	**0.001**	**0.042**	**0,000**	**0,000**
Enterococcus	0.19 [0.00;0.78]	0.15 [0.01;0.63]	0.765	0.938	0,550	0,740
Eubacterium	0.40 [0.11;1.03]	0.55 [0.20;1.41]	0.573	0.779	0,977	0,991
Eubacterium2	0.52 [0.03;1.97]	0.74 [0.06;1.38]	0.900	0.967	0,907	0,974
Faecalibacterium	3.09 [0.68;4.97]	3.16 [0.79;4.32]	0.803	0.957	0,373	0,600
Finegoldia	0.00 [0.00;0.07]	0.03 [0.00;0.20]	0.435	0.674	0,457	0,683
Flavonifractor	**0.00 [0.00;0.09]**	**0.00 [0.00;0.00]**	**0.025**	**0.075**	**0,000**	**0,000**
Fusobacterium	**0.00 [0.00;0.10]**	**0.00 [0.00;0.00]**	**0.025**	**0.075**	**0,000**	**0,000**
Gardnerella	**0.00 [0.00;0.00]**	**0.01 [0.00;0.51]**	**0.002**	**0.042**	**0,000**	**0,000**
Gemella	0.04 [0.00;0.26]	0.00 [0.00;0.07]	0.291	0.538	0,418	0,638
Gemmiger	0.61 [0.23;0.79]	0.56 [0.15;0.98]	0.708	0.919	0,816	0,940
Granulicatella	**0.06 [0.00;0.17]**	**0.00 [0.00;0.00]**	**0.001**	**0.042**	**0,000**	**0,000**
Haemophilus	**0.02 [0.00;0.17]**	**0.00 [0.00;0.00]**	**0.003**	**0.042**	**0,000**	**0,000**
Helicobacter	0.00 [0.00;0.31]	0.00 [0.00;0.30]	1.000	1.000	0,871	0,960
Herbaspirillum	0.00 [0.00;0.04]	0.02 [0.00;0.26]	0.139	0.346	0,188	0,419
Klebsiella	**0.00 [0.00;0.00]**	**0.00 [0.00;0.07]**	**0.004**	**0.042**	**0,000**	**0,000**
Kocuria	0.15 [0.03;0.24]	0.06 [0.00;0.14]	0.117	0.312	0,147	0,370
Lachnoclostridium	0.05 [0.00;0.10]	0.00 [0.00;0.03]	0.118	0.312	0,059	0,176
Lactobacillus	14.3 [3.48;24.4]	18.7 [2.31;24.8]	0.561	0.774	0,483	0,689
Lactococcus	0.03 [0.00;0.10]	0.03 [0.00;0.12]	0.894	0.967	0,901	0,974
Massilia	0.22 [0.04;23.9]	0.11 [0.00;0.25]	0.255	0.516	0,260	0,481
Megasphaera	0.01 [0.00;0.11]	0.02 [0.00;0.19]	0.824	0.967	0,975	0,991
Methylobacterium	0.57 [0.28;3.06]	0.33 [0.13;3.84]	0.533	0.772	0,825	0,940
Microbacterium	0.13 [0.00;0.49]	0.14 [0.02;0.39]	0.932	0.979	0,742	0,926

Micrococcus	0.15 [0.00;0.41]	0.01 [0.00;0.15]	0.163	0.389	0,067	0,194
Mitsuokella	0.00 [0.00;0.04]	0.02 [0.00;0.14]	0.197	0.451	0,219	0,454
Moraxella	0.02 [0.00;0.11]	0.10 [0.00;0.32]	0.242	0.510	0,177	0,406
Neisseria	0.07 [0.00;0.21]	0.00 [0.00;0.10]	0.165	0.389	0,203	0,441
Olsenella	**0.00 [0.00;0.00]**	**0.00 [0.00;0.07]**	**0.004**	**0.042**	**0,000**	**0,000**
Oscillibacter	**0.00 [0.00;0.00]**	**0.00 [0.00;0.07]**	**0.004**	**0.042**	**0,000**	**0,000**
Oxalicibacterium	0.00 [0.00;6.06]	0.00 [0.00;0.08]	0.862	0.967	0,781	0,926
Parabacteroides	0.49 [0.00;0.74]	0.30 [0.01;0.97]	0.966	0.989	0,940	0,985
Paracoccus	**0.00 [0.00;0.43]**	**0.00 [0.00;0.00]**	**0.006**	**0.048**	**0,000**	**0,000**
Parasutterella	**0.00 [0.00;0.04]**	**0.00 [0.00;0.00]**	**0.025**	**0.075**	**0,000**	**0,000**
Pelomonas	0.16 [0.00;0.58]	0.14 [0.00;0.40]	0.880	0.967	0,766	0,926
Phascolarctobacterium	**0.00 [0.00;0.12]**	**0.23 [0.08;0.47]**	**0.008**	**0.053**	**0,006**	**0,044**
Porphyromonas	0.00 [0.00;0.08]	0.00 [0.00;0.08]	0.747	0.938	0,630	0,809
Prevotella	9.29 [0.31;12.7]	12.3 [3.12;15.8]	0.280	0.538	0,174	0,406
Propionibacterium	3.09 [2.45;5.86]	1.76 [0.64;4.45]	0.096	0.270	0,162	0,391
Pseudoflavonifractor	0.00 [0.00;0.20]	0.00 [0.00;0.10]	1.000	1.000	0,788	0,926
Pseudomonas	**1.20 [0.59;6.56]**	**0.18 [0.06;0.80]**	**0.014**	**0.059**	**0,035**	**0,123**
Ralstonia	0.08 [0.00;0.28]	0.12 [0.00;0.25]	0.949	0.983	0,832	0,940
Rhizobium	**0.02 [0.00;0.20]**	**0.00 [0.00;0.00]**	**0.003**	**0.042**	**0,000**	**0,000**
Roseburia	0.23 [0.09;0.39]	0.56 [0.19;0.99]	0.061	0.177	0,244	0,471
Rothia	0.04 [0.00;0.16]	0.00 [0.00;0.06]	0.383	0.653	0,310	0,530
Ruminiclostridium	0.07 [0.00;0.22]	0.00 [0.00;0.06]	0.286	0.538	0,149	0,370
Ruminococcus	0.75 [0.14;1.53]	0.40 [0.26;1.10]	0.934	0.979	0,493	0,692
Ruminococcus2	0.89 [0.11;1.51]	0.47 [0.16;1.44]	0.803	0.957	0,933	0,985
Serratia	**0.00 [0.00;0.00]**	**0.00 [0.00;0.09]**	**0.010**	**0.053**	**0,000**	**0,000**
Shewanella	**0.00 [0.00;0.06]**	**0.00 [0.00;0.00]**	**0.025**	**0.075**	**0,000**	**0,000**
Sphingomonas	0.17 [0.00;0.57]	0.03 [0.00;0.23]	0.273	0.538	0,380	0,600
Staphylococcus	1.45 [0.62;2.67]	1.04 [0.51;1.56]	0.339	0.590	0,479	0,689
Streptococcus	2.08 [1.02;8.16]	1.18 [0.53;2.23]	0.244	0.510	0,317	0,530
Subdoligranulum	0.03 [0.00;0.15]	0.10 [0.01;0.34]	0.229	0.510	0,249	0,471
Sutterella	**0.00 [0.00;0.05]**	**0.00 [0.00;0.00]**	**0.012**	**0.054**	**0,000**	**0,000**
Veillonella	**0.00 [0.00;0.00]**	**0.00 [0.00;0.17]**	**0.004**	**0.042**	**0,000**	**0,000**
Vibrio	**0.00 [0.00;0.00]**	**0.00 [0.00;0.09]**	**0.010**	**0.053**	**0,000**	**0,000**

Supplementary Table 9. Microbial taxa comparisons between the endometrial and Fallopian tubes when considering only those patients with both tissue types of samples. With the bold are highlighted the bacterial taxa that differed significantly in their abundance in tissue type comparisons within whole groups (Supplementary Table 8). Relative abundances of detected genera are expressed as median and first and third quartiles (q1, q3) and were analyzed by non-paramatric Mann-Whitney *U* test. P-values were adjusted using the method of Benjamini and Hochberg to control for the multiple testing (False Discovery Rate, FDR).

	**Fallopian Tubes (n=7)**	**Uterus (n=7)**		
	Median [q1; q3]	Median [q1; q3]	P-value	Adjusted P-value
Aeromonas	0.08 [0.03;0.12]	0.00 [0.00;0.00]	0.009	0.197
Enhydrobacter	**0.10 [0.02;0.23]**	**0.00 [0.00;0.00]**	**0.009**	0.197
Granulicatella	**0.09 [0.04;0.16]**	**0.00 [0.00;0.00]**	**0.009**	0.197
Lachnoclostridium	0.05 [0.02;0.14]	0.00 [0.00;0.00]	0.009	0.197

Brachymonas	0.00 [0.00;0.00]	0.06 [0.00;0.15]	0.025	0.266
Klebsiella	**0.00 [0.00;0.00]**	**0.06 [0.00;0.30]**	**0.025**	0.266
Haemophilus	**0.03 [0.00;0.11]**	**0.00 [0.00;0.00]**	**0.025**	0.266
Phascolarctobacterium	0.00 [0.00;0.06]	0.12 [0.09;0.36]	0.046	0.266
Barnesiella	0.00 [0.00;0.00]	0.00 [0.00;0.08]	0.062	0.266
Olsenella	**0.00 [0.00;0.00]**	**0.00 [0.00;0.06]**	**0.062**	0.266
Serratia	0.00 [0.00;0.00]	0.00 [0.00;0.08]	0.062	0.266
Veillonella	**0.00 [0.00;0.00]**	**0.00 [0.00;0.28]**	**0.062**	0.266
Vibrio	0.00 [0.00;0.00]	0.00 [0.00;0.11]	0.062	0.266
Brachyspira	0.00 [0.00;0.08]	0.00 [0.00;0.00]	0.062	0.266
Burkholderia	0.00 [0.00;0.06]	0.00 [0.00;0.00]	0.062	0.266
Comamonas	0.00 [0.00;0.07]	0.00 [0.00;0.00]	0.062	0.266
Paracoccus	**0.00 [0.00;0.11]**	**0.00 [0.00;0.00]**	**0.062**	0.266
Parasutterella	0.00 [0.00;0.11]	0.00 [0.00;0.00]	0.062	0.266
Rhizobium	**0.00 [0.00;0.08]**	**0.00 [0.00;0.00]**	**0.062**	0.266
Shewanella	0.00 [0.00;0.07]	0.00 [0.00;0.00]	0.062	0.266
Bacteroides	0.39 [0.11;0.69]	1.13 [0.52;1.78]	0.11	0.422
Micrococcus	0.22 [0.10;0.23]	0.02 [0.00;0.07]	0.121	0.422
Gardnerella	**0.00 [0.00;0.00]**	**0.00 [0.00;0.01]**	**0.142**	0.422
Oscillibacter	**0.00 [0.00;0.00]**	**0.00 [0.00;0.02]**	**0.142**	0.422
Alistipes	**0.00 [0.00;0.07]**	**0.00 [0.00;0.00]**	**0.142**	0.422
Bifidobacterium	0.00 [0.00;0.02]	0.00 [0.00;0.00]	0.142	0.422
Fusobacterium	0.00 [0.00;0.04]	0.00 [0.00;0.00]	0.142	0.422
Sutterella	0.00 [0.00;0.03]	0.00 [0.00;0.00]	0.142	0.422
Streptococcus	2.05 [1.12;3.74]	0.93 [0.37;1.70]	0.142	0.422
Helicobacter	0.00 [0.00;0.61]	0.00 [0.00;0.00]	0.174	0.498
Acinetobacter	3.18 [1.31;11.7]	1.29 [0.76;3.25]	0.18	0.499
Bilophila	0.00 [0.00;0.09]	0.55 [0.00;0.64]	0.195	0.522
Ruminiclostridium	0.17 [0.00;0.39]	0.00 [0.00;0.09]	0.203	0.522
Moraxella	0.00 [0.00;0.07]	0.11 [0.05;0.15]	0.206	0.522
Staphylococcus	2.20 [0.95;2.92]	1.23 [0.65;1.46]	0.225	0.552
Gemmiger	0.63 [0.36;1.15]	0.46 [0.00;0.76]	0.245	0.585
Dorea	1.46 [0.38;1.90]	0.25 [0.13;1.05]	0.275	0.593
Butyricimonas	0.00 [0.00;0.00]	0.00 [0.00;0.20]	0.298	0.593
Campylobacter	0.00 [0.00;0.06]	0.09 [0.00;0.16]	0.306	0.593
Chryseobacterium	0.00 [0.00;0.00]	0.00 [0.00;0.00]	0.317	0.593
Flavonifractor	0.00 [0.00;0.00]	0.00 [0.00;0.00]	0.317	0.593
Rothia	0.08 [0.00;0.11]	0.00 [0.00;0.03]	0.322	0.593
Collinsella	0.20 [0.06;0.42]	0.09 [0.00;0.16]	0.327	0.593
Eubacterium2	0.54 [0.21;2.10]	0.06 [0.00;0.74]	0.327	0.593
Herbaspirillum	0.00 [0.00;0.02]	0.00 [0.00;0.33]	0.333	0.593
Roseburia	0.38 [0.07;0.44]	0.54 [0.12;0.90]	0.336	0.593
Arthrobacter	0.32 [0.07;0.56]	0.63 [0.28;1.19]	0.337	0.593
Corynebacterium	1.89 [0.35;2.72]	0.32 [0.22;1.67]	0.338	0.593
Faecalibacterium	4.59 [0.70;6.22]	0.84 [0.58;3.22]	0.338	0.593
Catenibacterium	2.23 [0.00;3.17]	0.31 [0.00;0.80]	0.352	0.605

Lactobacillus	21.0 [9.40;29.2]	13.5 [1.01;19.9]	0.406	0.672
Ruminococcus	1.99 [0.49;2.89]	0.36 [0.28;0.82]	0.406	0.672
Oxalicibacterium	0.00 [0.00;2.36]	0.05 [0.00;6.75]	0.479	0.768
Methylobacterium	0.89 [0.25;5.63]	4.89 [0.42;12.4]	0.482	0.768
Desulfovibrio	0.00 [0.00;0.04]	0.00 [0.00;0.14]	0.503	0.786
Actinomyces	0.00 [0.00;0.02]	0.00 [0.00;0.00]	0.534	0.807
Dolosigranulum	0.33 [0.00;0.71]	0.00 [0.00;0.26]	0.539	0.807
Clostridium	2.32 [0.99;4.15]	1.28 [0.36;2.09]	0.564	0.807
Eubacterium	0.70 [0.23;1.29]	0.50 [0.07;0.75]	0.564	0.807
Sphingomonas	0.19 [0.14;0.54]	0.08 [0.03;0.56]	0.564	0.807
Megasphaera	0.05 [0.00;0.08]	0.10 [0.00;0.25]	0.595	0.807
Parabacteroides	0.69 [0.00;1.21]	0.29 [0.00;0.45]	0.595	0.807
Gemella	0.07 [0.00;0.08]	0.00 [0.00;0.14]	0.633	0.807
Lactococcus	0.00 [0.00;0.20]	0.09 [0.00;0.26]	0.633	0.807
Microbacterium	0.00 [0.00;0.55]	0.16 [0.05;0.48]	0.642	0.807
Ruminococcus2	1.16 [0.06;1.77]	0.26 [0.14;0.66]	0.653	0.807
Blautia	0.25 [0.06;0.73]	0.37 [0.15;0.67]	0.655	0.807
Pseudomonas	0.76 [0.50;0.98]	0.25 [0.10;1.70]	0.655	0.807
Finegoldia	0.00 [0.00;0.03]	0.00 [0.00;0.00]	0.657	0.807
Porphyromonas	0.00 [0.00;0.03]	0.00 [0.00;0.00]	0.657	0.807
Subdoligranulum	0.00 [0.00;0.10]	0.06 [0.00;0.09]	0.733	0.870
Dialister	0.06 [0.00;0.23]	0.08 [0.01;0.23]	0.744	0.870
Massilia	0.05 [0.02;15.2]	0.27 [0.09;29.4]	0.747	0.870
Kocuria	0.14 [0.10;0.20]	0.15 [0.03;0.17]	0.748	0.870
Cloacibacterium	1.63 [0.00;1.82]	0.53 [0.15;0.93]	0.796	0.913
Anaerococcus	0.05 [0.00;0.19]	0.06 [0.01;0.13]	0.845	0.956
Bacillus	0.00 [0.00;0.14]	0.00 [0.00;0.08]	0.941	0.983
Pelomonas	0.00 [0.00;0.30]	0.00 [0.00;0.29]	0.944	0.983
Coprococcus	0.22 [0.00;0.57]	0.20 [0.01;0.48]	0.948	0.983
Ralstonia	0.07 [0.02;0.24]	0.10 [0.00;0.26]	0.948	0.983
Enterococcus	0.55 [0.30;1.05]	0.59 [0.04;1.61]	0.949	0.983
Prevotella	10.2 [0.27;14.2]	3.87 [1.71;14.5]	0.949	0.983
Propionibacterium	3.85 [2.53;7.31]	5.01 [1.18;12.1]	0.949	0.983
Mitsuokella	0.00 [0.00;0.08]	0.00 [0.00;0.04]	1.000	1.000
Neisseria	0.04 [0.00;0.07]	0.03 [0.00;0.09]	1.000	1.000
Pseudoflavonifractor	0.00 [0.00;0.06]	0.00 [0.00;0.01]	1	1.000

Supplementary Table 10. Shannon index comparisons of each pair of the tissue samples corresponding to their respective patient. Each value corresponds to the p value obtained using a Wilcoxon signed-rank test.

	**Patient 1**	**Patient 2**	**Patient 3**	**Patient 4**	**Patient 5**	**Patient 6**
**Patient 2**	0.7		-	-	-	-
**Patient 3**	0.875	0.875	-	-	-	-
**Patient 4**	0.7	1	0.875	-	-	-
**Patient 5**	1	0.7	0.875	0.7	-	-
**Patient 6**	1	0.7	1	0.7	0.875	-
**Patient 7**	0.875	0.7	1	0.7	0.7	0.7

Supplementary Table 11. OTUs number comparisons of each pair of the tissue samples corresponding to their respective patient. Each value corresponds to the p value obtained using a Wilcoxon signed-rank test.

	**Patient 1**	**Patient 2**	**Patient 3**	**Patient 4**	**Patient 5**	**Patient 6**
**Patient 2**	0.580	-	-	-	-	-
**Patient 3**	0.580	0.737	-	-	-	-
**Patient 4**	1.000	0.580	0.580	-	-	-
**Patient 5**	0.737	0.580	0.580	0.580	-	-
**Patient 6**	0.580	0.580	0.580	0.580	0.580	-
**Patient 7**	0.737	0.580	0.737	1.000	0.580	0.580
